# Supplementary material for: Identification of Freezing-Responsive microRNAs and Their Targets in Chinese Jujube by Small RNA and Degradome Sequencing
Source: Int J Mol Sci. 2026 Sep 4;27(17):7912. doi: 10.3390/ijms27177912 (PMC13566890; doi:10.3390/ijms27177912)
Supplement: Supplementary file 1 [file ijms-27-07912-s001.zip › Supplementary Material S2/novel_miR_52.pdf]

Provisional ID	:	novel_miR_52
Score total	:	71764.6
Score for star read(s)	:	3.9
Score for read counts	:	71758
Score for mfe	:	1
Score for randfold	:	1.6
Score for cons. seed	:	
Total read count	:	140762
Mature read count	:	140253
Loop read count	:	1
Star read count	:	508

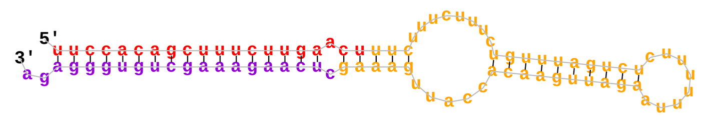

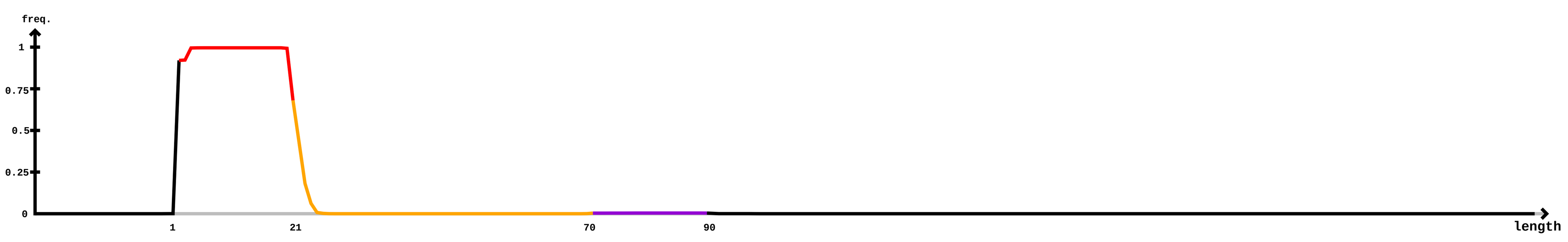

Mature Star

```
5'- uuugcaaguccuggucaugcuuuuccacagcuuucuugaacuuucuuucuuucuguuuagucucuuuuuuaagauugaacaccauugaaagcucaagaaagcugugggagaauauggcaaauuugggauuuuaauuuuuuuauuuuuguuuuuauuuuuuuauuuuuccuucaacuacucuucagcugaugaugauguugcacacagaaagaaagaaagaaagaaagaaagagagaucucagaagcuaaau -3' obs
    uuugcaaguccuggucaugcuuuuccacagcuuucuugaacuuucuuucuuucuguuuagucucuuuuuuaagauugaacaccauugaaagcucaagaaagcugugggagaauauggcaaauuugggauuuuaauuuuuuuauuuuuguuuuuauuuuuuuauuuuuccuucaacuacucuucagcugaugaugauguugcacacagaaagaaagaaagaaagaaagaaagagagaucucagaagcuaaau exp
    ............(((((.((((((((((((((((((.(((((........(((((((((........))))))))).....))))).)))))))))))))))))).))))))...((((((((((..((((((.(((((.(((((.((((((..........((((...((.(((.....))).)).))))......)))))).))))).))))).)))))))))))))))))........
```

read	reads	mm	sample
....................uuuuccacagcuuucuugaac	3	0	LT-1
....................uuuuccacagcuuucuugaac	4	0	JCK-1
....................uuuuccacagcuuucuugaac	3	0	LT-3
....................uuuuccacagcuuucuugaac	2	0	JT-2
....................uuuuccacagcuuucuugaac	3	0	JT-3
....................uuuuccacagcuuucuugaac	3	0	LCK-3
....................uuuuccacagcuuucuugaac	7	0	JCK-3
....................uuuuccacagcuuucuugaac	6	0	JCK-2
....................uuuuccacagcuuucuugaacu	4	0	JCK-2
....................uNuuccacagcuuucuugaacu	1	1	JCK-3
....................uuuuccacagcuuucuugaacu	1	0	JCK-3
.....................uuuccacagcuuucuuga	2	0	JCK-1
.....................Guuccacagcuuucuugaacu	2	1	LT-3
.....................uuuccacagcuuucuugaacu	1	0	LT-1
.....................uuuccacagcuuucuugaacu	1	0	JCK-2
.....................Auuccacagcuuucuugaacu	1	1	JCK-3
.....................uuuccacagcuuucuugaacu	2	0	LCK-3
.....................Auuccacagcuuucuugaacu	2	1	JCK-2
.....................uuuccacagcuuucuugaacu	1	0	JCK-3
.....................Cuuccacagcuuucuugaacuu	1	1	LT-2
.....................Cuuccacagcuuucuugaacuu	3	1	LT-3
.....................Cuuccacagcuuucuugaacuu	3	1	LT-1
.....................Cuuccacagcuuucuugaacuu	2	1	JCK-1
.....................Cuuccacagcuuucuugaacuu	3	1	JCK-2
.....................Cuuccacagcuuucuugaacuuu	3	1	LCK-3
.....................Cuuccacagcuuucuugaacuuu	1	1	LT-1
.....................Cuuccacagcuuucuugaacuuu	1	1	LT-3
.....................Cuuccacagcuuucuugaacuuu	2	1	JCK-1
......................uuccacagcuuucuugCa	1	1	LT-2
......................uuccacagcuuucuugUa	19	1	LCK-3
......................uuccacagcuuucuugaU	1	1	JCK-1
......................uuccacagcuuucuuUaa	1	1	LT-1
......................uuccacagcuuucuugUa	1	1	LT-1

Mature Star

Sequence			
uuugcaaguccuggucaugcuuuuccacagcuuucuugaacuuucuuucuuucuguuuagucucuuuuuuaagauugaacaccauugaaagcucaagaaagcugugggagaauauggcaaauuugggauuuuaauuuuuuuauuuuuguuuuuauuuuuuauuuuuccuucaacuacucuucagcugaugaugauguugcacacagaaagaaagaaagaaagaaagaaagagagaucucagaagcuaaau			
......................uuccacagcuuucuugaa	43	0	LT-2
......................uuccacagcuuucuugaa	9	0	JCK-3
......................uuccacagcuuucuugaa	31	0	LT-1
......................uuccacagcuuucuugaa	52	0	LT-3
......................uuccacagcuuucuugaa	46	0	LCK-1
......................uuccacagcuuucuugaU	3	1	LT-2
......................uuccacagcuuucuugaU	4	1	LCK-3
......................uuccacagcuuucuugaa	15	0	JT-3
......................uuccacagcuuucuugUa	4	1	LT-3
......................uuccacagcuuucuugaa	16	0	JCK-1
......................uuccacagcuuucuugaa	5	0	JT-2
......................uuccacagcuuucuugUa	15	1	LT-2
......................uuccacagcuuucuugaC	1	1	LT-3
......................uuccacagcuuucuugGa	1	1	LCK-2
......................uuccacagcuuucuugaU	11	1	LT-1
......................uuccacagcuuucuugaC	1	1	JCK-3
......................uuccacagcuuucuugaa	53	0	LCK-2
......................uuccacagcuuucuugaa	15	0	JCK-2
......................uuccacagcuuucuugUa	2	1	JCK-3
......................uuccacagcuuucuGgaa	1	1	LCK-1
......................uuccacagcuuucuugaC	4	1	LT-2
......................uuccacagcuuucuugaa	43	0	LCK-3
......................uuccaNagcuuucuugaa	1	1	LCK-1
......................uuccacagcuuucuugaU	1	1	JCK-2
......................uuccacagcuuucuugUa	5	1	JT-1
......................uuccacagcuuucuugaU	14	1	LT-3
......................uuccacagcuuucuugaCc	7	1	JCK-2
......................uuccacagcuuucCugaac	1	1	JCK-3
......................uuccacagcuuucCugaac	1	1	LT-2
......................uuccacCgcuuucuugaac	12	1	LT-1
......................uuccacagcuuucuugaac	2180	0	JCK-1
......................uCccacagcuuucuugaac	1	1	LCK-1
......................uuccacagcuuuNuugaac	1	1	LCK-1
......................uuccacagcuuucuugGac	1	1	JCK-3
......................uuUcacagcuuucuugaac	1	1	JCK-3
......................uuccacagcuuucuugaCc	2	1	LCK-2
......................uuccacagcuuucuugaCc	8	1	LCK-3
......................uuccaAagcuuucuugaac	1	1	JCK-2
......................uNccacagcuuucuugaac	2	1	JCK-3
......................uuccacagcuuucuugaUc	3	1	LT-2
......................uuccacagcuuucuugaac	8415	0	LCK-3
......................uuccacagcuuuUuugaac	4	1	JCK-1
......................uuccacagcuuucuugaGc	1	1	JCK-1
......................uuccacCgcuuucuugaac	2	1	JCK-1
......................uuccacagcuuucCugaac	3	1	JCK-2
......................uuccacagcuuucuugCac	11	1	LT-3
......................uuccacagcuuucuugCac	5	1	JCK-2
......................Cuccacagcuuucuugaac	1	1	JCK-3
......................uuccacagcuCucuugaac	1	1	LT-1
......................uCccacagcuuucuugaac	1	1	JCK-2
......................uuccacagcuuCcuugaac	3	1	LCK-3
......................uuccaAagcuuucuugaac	3	1	LT-3
......................Cuccacagcuuucuugaac	3	1	LT-3
......................uuccacagcuuucCugaac	2	1	LCK-3
......................uuUcacagcuuucuugaac	6	1	JCK-1
......................uuccacCgcuuucuugaac	2	1	LCK-2
......................uuccacagcuuucuugaCc	5	1	LT-1
......................uuccacagcuuucuugaac	1676	0	LCK-1
......................uuccacagcuuuUuugaac	3	1	LT-1
......................uuccacagcuuucuugaac	8638	0	LT-3
......................uuccacagcuuCcuugaac	3	1	JCK-1
......................uuccGcagcuuucuugaac	1	1	LT-1
......................uuccacagcuuucuuUaac	1	1	JT-1
......................uuccacagcuuucuugUac	3	1	LCK-3
......................uuccacagUuuucuugaac	1	1	LT-3
......................uuccCcagcuuucuugaac	2	1	LCK-1
......................uuccacagcuuuUuugaac	6	1	LT-2
......................uuccacCgcuuucuugaac	2	1	JT-1
......................uucUacagcuuucuugaac	2	1	JCK-1
......................uNccacagcuuucuugaac	5	1	JCK-2

```
                      Mature                                                                     Star
uuugcaaguccuggucaugcuuuuccacagcuuucuugaacuuucuuucuuucuguuuagucucuuuuuuaagauugaacaccauugaaagcucaagaaagcuguggagaauauggcaaauuugggauuuuaauuuuuuuauuuuuguuuuuauuuuuuauuuuuccuucaacuacucuucagcugaugaugauguugcacacagaaagaaagaaagaaagaaagaaagagagaucucagaagcuaaau
......................uuccacagcuuucuugaac................................................................................................................................................................................................................  3571  0  JCK-2
......................uuccacaAcuuucuugaac................................................................................................................................................................................................................  1     1  JCK-3
......................uuccacaAcuuucuugaac................................................................................................................................................................................................................  2     1  LCK-3
......................uuccCcagcuuucuugaac................................................................................................................................................................................................................  2     1  JT-1
......................uuccacagcuuucuuAaac................................................................................................................................................................................................................  4     1  LT-2
......................uuccacagcuuucuugCac................................................................................................................................................................................................................  1     1  LCK-2
......................uuccacagcuuucuugaGc................................................................................................................................................................................................................  1     1  LCK-3
......................uuccCcagcuuucuugaac................................................................................................................................................................................................................  1     1  JCK-1
......................uuccacagcuuucuugaUc................................................................................................................................................................................................................  10    1  LCK-3
......................uuccacagcuuucuugaac................................................................................................................................................................................................................  1739  0  LCK-2
......................uuccacagcuuucuugaaU................................................................................................................................................................................................................  24    1  JCK-1
......................uuccacagcuuucuugaac................................................................................................................................................................................................................  411   0  JT-2
......................uuccacagcuGucuugaac................................................................................................................................................................................................................  3     1  JCK-1
......................uuccacaAcuuucuugaac................................................................................................................................................................................................................  1     1  LT-3
......................uNccacagcuuucuugaac................................................................................................................................................................................................................  1     1  LCK-2
......................uuccCcagcuuucuugaac................................................................................................................................................................................................................  16    1  LT-3
......................uuccacagcuuucuugaUc................................................................................................................................................................................................................  3     1  JT-1
......................uuccacagcuuucuugCac................................................................................................................................................................................................................  4     1  LT-1
......................uuccCcagcuuucuugaac................................................................................................................................................................................................................  13    1  LCK-3
......................uuccacagcuuucuugCac................................................................................................................................................................................................................  3     1  JT-1
......................uuccacCgcuuucuugaac................................................................................................................................................................................................................  2     1  JCK-3
......................uuccacagcuuucuugaCc................................................................................................................................................................................................................  1     1  LCK-1
......................uuccacagcCuucuugaac................................................................................................................................................................................................................  1     1  JCK-1
......................uuccaNagcuuucuugaac................................................................................................................................................................................................................  3     1  LCK-1
......................uuccacCgcuuucuugaac................................................................................................................................................................................................................  16    1  LT-3
......................uuccacagcuuuUuugaac................................................................................................................................................................................................................  1     1  LCK-2
......................uuccacagcuuuAuugaac................................................................................................................................................................................................................  1     1  LT-2
......................uuccacGgcuuucuugaac................................................................................................................................................................................................................  2     1  JCK-2
......................uuccacagcuuucuGgaac................................................................................................................................................................................................................  1     1  JCK-2
......................uuccacagcuuucuugUac................................................................................................................................................................................................................  1     1  LT-3
......................uuccacagcCuucuugaac................................................................................................................................................................................................................  1     1  LT-2
......................uuccCcagcuuucuugaac................................................................................................................................................................................................................  33    1  LT-2
......................uuccacagcuuucuGgaac................................................................................................................................................................................................................  2     1  LCK-1
......................uuccacagcCuucuugaac................................................................................................................................................................................................................  5     1  LT-3
......................uuccacagcuuCcuugaac................................................................................................................................................................................................................  2     1  JCK-3
......................uuccacagcuuucuugaaU................................................................................................................................................................................................................  98    1  LT-3
......................Cuccacagcuuucuugaac................................................................................................................................................................................................................  1     1  LT-2
......................Cuccacagcuuucuugaac................................................................................................................................................................................................................  4     1  LT-1
......................uNccacagcuuucuugaac................................................................................................................................................................................................................  3     1  JT-1
......................uuccacagcuuuAuugaac................................................................................................................................................................................................................  2     1  LT-1
......................uCccacagcuuucuugaac................................................................................................................................................................................................................  2     1  LT-3
......................uuccacagcuuucCugaac................................................................................................................................................................................................................  2     1  LT-1
......................uuUcacagcuuucuugaac................................................................................................................................................................................................................  1     1  LT-1
......................uuccacagcuuucuugaaU................................................................................................................................................................................................................  37    1  LT-1
......................uuccacaAcuuucuugaac................................................................................................................................................................................................................  5     1  LT-1
......................uuccacagcuuucuugCac................................................................................................................................................................................................................  5     1  LT-2
......................uuccacCgcuuucuugaac................................................................................................................................................................................................................  10    1  LT-2
......................uuccacagcuuucuugaac................................................................................................................................................................................................................  1423  0  JT-1
......................uuccacagcuuucuugaCc................................................................................................................................................................................................................  1     1  JCK-3
......................uuccacagcuuucuugaac................................................................................................................................................................................................................  542   0  JT-3
......................uNccacagcuuucuugaac................................................................................................................................................................................................................  1     1  JCK-1
......................uuccacagcuuucuugaCc................................................................................................................................................................................................................  10    1  LT-3
......................uuccacCgcuuucuugaac................................................................................................................................................................................................................  9     1  JCK-2
......................uuccacagcCuucuugaac................................................................................................................................................................................................................  1     1  JCK-2
......................uuccacaAcuuucuugaac................................................................................................................................................................................................................  2     1  LT-2
......................uCccacagcuuucuugaac................................................................................................................................................................................................................  1     1  JCK-1
......................uuccaUagcuuucuugaac................................................................................................................................................................................................................  1     1  JCK-2
......................uuccCcagcuuucuugaac................................................................................................................................................................................................................  4     1  JCK-2
......................uuccacagcuGucuugaac................................................................................................................................................................................................................  1     1  LT-1
......................uuccCcagcuuucuugaac................................................................................................................................................................................................................  2     1  JCK-3
......................uuUcacagcuuucuugaac................................................................................................................................................................................................................  2     1  JCK-2
......................uuGcacagcuuucuugaac................................................................................................................................................................................................................  3     1  LT-2
......................uuccacagcuuCcuugaac................................................................................................................................................................................................................  2     1  LT-1
......................uuccacagcuuucuugaCc................................................................................................................................................................................................................  13    1  LT-2
......................uuccacagcuuucuugaac................................................................................................................................................................................................................  2667  0  JCK-3
......................Cuccacagcuuucuugaac................................................................................................................................................................................................................  2     1  JT-1
......................uuccacagcuuucuuAaac................................................................................................................................................................................................................  1     1  JCK-1
......................uuccacaAcuuucuugaac................................................................................................................................................................................................................  1     1  JCK-2
......................uucUacagcuuucuugaac................................................................................................................................................................................................................  1     1  JCK-3
......................uuccCcagcuuucuugaac................................................................................................................................................................................................................  1     1  JT-3
```

Mature **Star**

uuugcaaguccuggucaugcuuuuccacagcuuucuugaacuuucuuucuuucuguuuagucucuuuuuuaagauugaacaccauugaaagcucaagaaagcugugggagaauauggcaaauuugggauuuuaauuuuuuuauuuuuguuuuuauuuuuuauuuuuccuucaacuacucuucagcugaugaugauguugcacacagaaagaaagaaagaaagaaagaaagagagaucucagaagcuaaau

uuccacagcuuucGugaac	3	1	LCK-1
uuccCcagcuuucuugaac	1	1	LCK-2
uucNacagcuuucuugaac	1	1	JCK-1
uuccacagcuuuUuugaac	8	1	JCK-2
uucAacagcuuucuugaac	1	1	LT-2
uuccacagcuuucuugCac	1	1	LCK-1
uuAcacagcuuucuugaac	1	1	JCK-1
uuccacagcuuucuugaUc	2	1	LT-3
uuccacagcuuNcuugaac	2	1	JCK-1
uuUcacagcuuucuugaac	1	1	LT-3
uuccaUagcuuucuugaac	2	1	LT-3
uucUacagcuuucuugaac	4	1	JCK-2
uuccacagcuuucuugaac	5694	0	LT-2
Cuccacagcuuucuugaac	3	1	LCK-3
uuccacagcuuucuugaaU	64	1	LT-2
uuccacagAuuucuugaac	1	1	LT-2
uuccacagcuuucNugaac	6	1	LCK-1
uuNcacagcuuucuugaac	14	1	LT-2
uuccacagcuuucuugCac	1	1	JCK-3
uuccacagcuuucuugaGc	1	1	LT-3
uuccacagcuuuAuugaac	2	1	LCK-3
uuccacagUuuucuugaac	5	1	LT-1
uuAcacagcuuucuugaac	1	1	LT-3
uuccacagcuuucuugaaU	28	1	JCK-3
uuccaAagcuuucuugaac	1	1	LT-2
uuccaAagcuuucuugaac	1	1	LCK-3
uuccacagcuuucuugaaU	24	1	LCK-2
uuccacagcuuucuugaaU	26	1	LCK-1
uuccacagcuuucuugaCc	1	1	JT-1
uuccacagcuuucuugaUc	2	1	JCK-1
uuccacagcuuucuCgaac	1	1	LT-1
uuccacagcuCucuugaac	2	1	JCK-2
uuccCcagcuuucuugaac	12	1	LT-1
uuccacagcuuCcuugaac	3	1	JCK-2
uuccacagUuuucuugaac	4	1	LCK-3
uuccacagcuuucuugaac	6061	0	LT-1
uuccacagcuuucuNgaac	4	1	LCK-1
uuccacagcCuucuugaac	7	1	LT-1
uuccacagcuuucuugaaU	53	1	JCK-2
uuccacaUcuuucuugaac	2	1	JCK-2
uuccacagcuuuUuugaac	2	1	JCK-3
uuccacagcuuucuugCac	3	1	LCK-3
uuNcacagcuuucuugaac	1	1	LCK-1
uuccacagcuuucuugCac	2	1	JCK-1
uuccacCgcuuucuugaac	11	1	LCK-3
uuccaUagcuuucuugaac	1	1	LT-2
uuccacagcuuucuugaaU	103	1	LCK-3
uuccacagUuuucuugaac	3	1	LT-2
uuccacCgcuuucuugaac	1	1	JT-2
uuccaAagcuuucuugaac	1	1	JCK-1
uuccacagcuuucuugaacA	20	1	JT-1
uuccacagcCuucuugaacu	1	1	JCK-1
uuccacagcuuucuugaCcu	8	1	LT-3
uuccacagcuuucuuCaacu	1	1	JCK-1
uuccCcagcuuucuugaacu	4	1	JCK-1
uuccacagcuuucuugaacG	2	1	LT-1
uuccCcagcuuucuugaacu	8	1	LT-3
uuccaAagcuuucuugaacu	1	1	JCK-2
uuccacCgcuuucuugaacu	13	1	LT-2
uuccacagcCuucuugaacu	5	1	LT-1
uuccacagcuuucuugaacG	1	1	JT-1
uuccacagcuuucuugaaUu	45	1	JT-2
uuccacagcuuucuugaCcu	11	1	LCK-3
uuccacagcuuucuugaacC	5	1	JCK-3
uuccacCgcuuucuugaacu	3	1	JCK-3
uuccacagcuuucuCgaacu	1	1	JCK-1
uuccacagUuuucuugaacu	1	1	LT-1
uuccacagcCuucuugaacu	1	1	LT-3
uuccacagcuuCcuugaacu	2	1	LT-2
uuccacagcuuucuugaCcu	3	1	LCK-1

Mature   Star

uuugcaaguccuggucaugcuuuuccacagcuuucuugaacuuucuuucuuucuguuuagucucuuuuuuaagauugaacaccauugaaagcucaagaaagcugugggagaauauggcaaauuugggauuuuaauuuuuuuauuuuuguuuuuauuuuuuauuuuuccuucaacuacucuucagcugaugaugauguugcacacagaaagaaagaaagaaagaaagaaagagagaucucagaagcuaaau

......................uuccacagcuuucCugaacu	1	1	JT-1
......................uuccacagcuuucuugaGcu	2	1	LCK-3
......................uuccacagcuuucuugaacu	3389	0	JCK-2
......................uuccacagcuuCcuugaacu	2	1	LT-3
......................uuccacagcuuucuugaacu	2316	0	LCK-1
......................uuccacaUcuuucuugaacu	1	1	JCK-1
......................uuccacagcCuucuugaacu	3	1	JCK-2
......................uuccacagcuCucuugaacu	3	1	LT-1
......................uCccacagcuuucuugaacu	2	1	JCK-1
......................uuccacagcuuucuuAaacu	1	1	JCK-1
......................uuccacagcuuucuugaaUu	192	1	LCK-3
......................uuccacagcuuucuugaacA	87	1	LT-1
......................uuccacagcuuuNuugaacu	1	1	LCK-1
......................uuccacagcCuucuugaacu	1	1	JT-3
......................uuccacagcuuucuugaacC	3	1	JT-3
......................uuccacagcuuucuugaacG	8	1	LT-2
......................uuccaAagcuuucuugaacu	2	1	JCK-1
......................uuccacagcuuucuugaaUu	52	1	JCK-3
......................uuccacagcuuucCugaacu	1	1	LT-1
......................uuccacagcuuucuugaacu	10755	0	LCK-3
......................uuccacagcuuucuugaacA	48	1	JCK-1
......................uuccacagcuuuAuugaacu	1	1	LT-2
......................uNccacagcuuucuugaacu	4	1	JCK-1
......................uuccacagcuuucuugGacu	1	1	LT-2
......................uuUcacagcuuucuugaacu	1	1	JCK-3
......................uuccacagcuuucuugaaUu	50	1	JT-3
......................uuccacagcuuucuugaUcu	2	1	LT-1
......................uuccacCgcuuucuugaacu	3	1	JCK-1
......................uuccacagcuuucuCgaacu	1	1	LT-3
......................uuccacagcCuucuugaacu	7	1	LT-2
......................uNccacagcuuucuugaacu	2	1	LCK-1
......................uuccaNagcuuucuugaacu	3	1	LCK-1
......................Cuccacagcuuucuugaacu	1	1	LT-2
......................uuccacagcuuucuugCacu	10	1	LT-1
......................uuccacagcuuucuugaaAu	6	1	LCK-3
......................uuccacagcuuucCugaacu	1	1	JCK-2
......................uuccacagcuuucCugaacu	2	1	LCK-3
......................uuccacagcuuucuugaacN	2	1	JCK-1
......................uuccGcagcuuucuugaacu	1	1	LCK-3
......................uuccacagcuuucuugaCcu	1	1	JT-2
......................uuccacagcuuucuugaacG	2	1	JCK-2
......................uuccacagcuuucuugaacC	7	1	JCK-1
......................uuccacagcuuucuugCacu	3	1	JCK-2
......................uuccacagcuuucuugaaUu	72	1	JCK-1
......................uuccacCgcuuucuugaacu	2	1	LCK-1
......................uuccacagcuGucuugaacu	1	1	LT-2
......................uuccacCgcuuucuugaacu	3	1	LCK-2
......................uuccacCgcuuucuugaacu	1	1	JT-1
......................uuccaAagcuuucuugaacu	1	1	LCK-1
......................uuccacagcuuucuugaaAu	2	1	LT-1
......................uuccacagcuuucuugaacu	2309	0	JCK-3
......................uuccacagUuuucuugaacu	1	1	LCK-3
......................uuccacGgcuuucuugaacu	1	1	LCK-3
......................uuccaAagcuuucuugaacu	3	1	LT-1
......................uuccacagcuuucuugaacA	3	1	JT-2
......................uCccacagcuuucuugaacu	2	1	JCK-2
......................uuccacagcuuucuugaacC	2	1	JCK-2
......................uuccacCgcuuucuugaacu	9	1	LCK-3
......................uuccacagcuuucuugCacu	3	1	JCK-1
......................uuccacagcuuucuugaaAu	1	1	JCK-3
......................uuccacagcuuucuugaaUu	132	1	LT-1
......................uuccacagcuuucGugaacu	1	1	LCK-2
......................uuccacGgcuuucuugaacu	2	1	JT-3
......................uuccacagcuuucuugGacu	8	1	LCK-3
......................uuccacagcuuucuugaaUu	35	1	LCK-2
......................uuccCcagcuuucuugaacu	2	1	JT-3
......................uuccacagUuuucuugaacu	1	1	JT-1
......................uuccacagcuuCcuugaacu	1	1	LCK-3
......................uuccacagcuuucuugaacC	8	1	JT-1
......................uuccacagcuuucuugaacu	2326	0	LCK-2

Mature | Star

uuugcaaguccuggucaugcuuuuccacagcuuucuugaacuuucuuucuuucuguuuagucucuuuuuuaagauugaacaccauugaaagcucaagaaagcugugggagaauauggcaaauuugggauuuuaauuuuuuuauuuuuguuuuauuuuuuauuuuuccuucaacuacucuucagcugaugaugauguugcacacagaaagaaagaaagaaagaaagaaagagagaucucagaagcuaaau

......................uuccacagUuuucuugaacu........	1	1	JCK-2
......................uuccacagcuuucuugCacu........	6	1	LT-3
......................uuNcacagcuuucuugaacu........	33	1	LT-2
......................uuccacagcuuucuugaNcu........	1	1	LCK-1
......................uuccacagcuuucuugaCcu........	1	1	JCK-3
......................uuccacCgcuuucuugaacu........	6	1	JCK-2
......................uuccacagcuuuAuugaacu........	4	1	LCK-3
......................uuccacagcuuucuuAaacu........	1	1	LT-2
......................uuccacagcuuucuugaacA........	11	1	JT-3
......................uuGcacagcuuucuugaacu........	5	1	LT-2
......................uuccacaAcuuucuugaacu........	1	1	LCK-2
......................uuccacagcuuucuugaCcu........	3	1	JT-1
......................uuccacaAcuuucuugaacu........	4	1	LCK-3
......................uuccacagcuuucuGgaacu........	4	1	LCK-1
......................uuccaUagcuuucuugaacu........	3	1	JCK-2
......................uuccaAagcuuucuugaacu........	1	1	JCK-3
......................Cuccacagcuuucuugaacu........	2	1	JCK-3
......................uuccacagcuuucuugaacA........	196	1	LCK-3
......................uuccacagcuCucuugaacu........	1	1	LCK-3
......................uuccacagcuuucGugaacu........	1	1	LCK-1
......................uuccacagcuuCcuugaacu........	1	1	JCK-2
......................uuccacagcuuucuugaacu........	2125	0	JCK-1
......................uuccacagcuuucuugaacA........	172	1	LT-3
......................uuccacagcuuucuugGacu........	1	1	LT-3
......................uuccacagcuuucuugaGcu........	3	1	JCK-1
......................uuccacagcuuuAuugaacu........	2	1	JCK-3
......................uuccacagcuCucuugaacu........	1	1	JT-1
......................uNccacagcuuucuugaacu........	1	1	JCK-2
......................Cuccacagcuuucuugaacu........	1	1	JCK-2
......................uuccacagcuuNcuugaacu........	3	1	JCK-1
......................uuccacagcuuucuugaacu........	731	0	JT-3
......................uuccacCgcuuucuugaacu........	4	1	LT-1
......................uuccacagcuuucuugaacC........	2	1	LCK-1
......................uuccCcagcuuucuugaacu........	1	1	JCK-2
......................uuccacagcuuucuugaaNu........	1	1	LT-1
......................uuccCcagcuuucuugaacu........	8	1	LCK-3
......................uuccacagcuuucCugaacu........	1	1	LT-3
......................uCccacagcuuucuugaacu........	1	1	LT-1
......................uuccacagcuuucuugCacu........	2	1	LCK-1
......................uuccacagcuuucuugaacC........	18	1	LT-3
......................uuUcacagcuuucuugaacu........	4	1	JCK-2
......................uuccaAagcuuucuugaacu........	3	1	LT-3
......................uuccacaAcuuucuugaacu........	1	1	LT-1
......................uuccacagcuuucuugaacA........	57	1	JCK-2
......................uuccacagcuuucuugaacA........	2	1	LCK-1
......................uuccacagcuuucuugaacu........	544	0	JT-2
......................uuccacagcuuucuugaaAu........	1	1	JCK-1
......................uuccacagcuuucuugaUcu........	2	1	LT-2
......................uuccacagcuuucuCgaacu........	1	1	LT-2
......................uuccacagcuuucNugaacu........	5	1	LCK-1
......................uuccacagcuuucuugaaUu........	154	1	LT-2
......................uuccacagcuGucuugaacu........	1	1	JCK-1
......................uucUacagcuuucuugaacu........	1	1	JCK-1
......................uCccacagcuuucuugaacu........	1	1	LCK-3
......................uuccacagcuuucuugaacu........	6887	0	LT-2
......................uuccacagcuuucuugaacu........	7087	0	LT-3
......................uuccacagcuuucuugaaUu........	24	1	LCK-1
......................uucUacagcuuucuugaacu........	3	1	LCK-3
......................uuccacagcCuucuugaacu........	1	1	JT-2
......................Cuccacagcuuucuugaacu........	4	1	LCK-3
......................Cuccacagcuuucuugaacu........	2	1	LCK-1
......................uuccacagcuuucuuAaacu........	3	1	LCK-3
......................uuccacagcuuuAuugaacu........	2	1	LT-3
......................uuNcacagcuuucuugaacu........	2	1	LCK-3
......................uuccacagcuuucuugaCcu........	1	1	LCK-2
......................uuccacagcuuucuugaCcu........	3	1	JCK-1
......................uuccCcagcuuucuugaacu........	2	1	JT-1
......................uuccacagcuuNcuugaacu........	1	1	JT-2
......................uuccacagcuuuUuugaacu........	1	1	LT-3
......................Cuccacagcuuucuugaacu........	1	1	LT-3

Mature **Star**

```
uuugcaaguccuggucaugcuuuuccacagcuuucuugaacuuucuuucuuucuguuuagucucuuuuuuaagauugaacaccauugaaagcucaagaaagcugugggagaauauggcaaauuugggauuuuaauuuuuuuauuuuuguuuuuauuuuuuauuuuuccuucaacuacucuucagcugaugaugauguugcacacagaaagaaagaaagaaagaaagaaagagagaucucagaagcuaaau
......................uuccacaAcuuucuugaacu...........................................................................................................................................................................................   1      1   JT-1
......................uuccacagcuuucuugaacC...........................................................................................................................................................................................   3      1   JT-2
......................uuccacagcuuucuugaacC...........................................................................................................................................................................................   9      1   LT-1
......................uuccacagcuuucuugaaAu...........................................................................................................................................................................................   2      1   LT-3
......................uucUacagcuuucuugaacu...........................................................................................................................................................................................   3      1   LT-2
......................uuccacagcuuucuGgaacu...........................................................................................................................................................................................   1      1   JT-1
......................uuccacagcuuucuugaNcu...........................................................................................................................................................................................   2      1   JCK-3
......................uuccacagcuuucuugaCcu...........................................................................................................................................................................................   25     1   LT-2
......................uuccCcagcuuucuugaacu...........................................................................................................................................................................................   20     1   LT-2
......................uCccacagcuuucuugaacu...........................................................................................................................................................................................   1      1   LT-3
......................uuccacagcuuucuugaacu...........................................................................................................................................................................................   2055   0   JT-1
......................uuccCcagcuuucuugaacu...........................................................................................................................................................................................   1      1   LCK-1
......................uuccacagcuuuUuugaacu...........................................................................................................................................................................................   2      1   LT-1
......................uuccUcagcuuucuugaacu...........................................................................................................................................................................................   1      1   JCK-2
......................uuccacagcuuucuugaCcu...........................................................................................................................................................................................   3      1   JCK-2
......................uuccacagcuuucuugaacC...........................................................................................................................................................................................   39     1   LT-2
......................uuccacagcuuNcuugaacu...........................................................................................................................................................................................   3      1   JT-1
......................uuccacagcuuucuugaacu...........................................................................................................................................................................................   4415   0   LT-1
......................uuccacagcuuucuNgaacu...........................................................................................................................................................................................   7      1   LCK-1
......................uuccacagcuuucuugaacC...........................................................................................................................................................................................   41     1   LCK-3
......................uuccacagcuuucuugaaUu...........................................................................................................................................................................................   18     1   JT-1
......................uuccacagcuuucuugCacu...........................................................................................................................................................................................   2      1   JCK-3
......................uuccacaAcuuucuugaacu...........................................................................................................................................................................................   1      1   LT-2
......................uuccacagcuuucuugaaUu...........................................................................................................................................................................................   111    1   JCK-2
......................uuccacagcuuucuugCacu...........................................................................................................................................................................................   10     1   LCK-3
......................uNccacagcuuucuugaacu...........................................................................................................................................................................................   1      1   JT-1
......................uuccacagcuuucuugaCcu...........................................................................................................................................................................................   1      1   LT-1
......................uuccacaAcuuucuugaacu...........................................................................................................................................................................................   1      1   LT-3
......................uuccacagcuuucuugCacu...........................................................................................................................................................................................   2      1   LCK-2
......................uuccacagcuCucuugaacu...........................................................................................................................................................................................   2      1   LT-2
......................uucNacagcuuucuugaacu...........................................................................................................................................................................................   2      1   JCK-1
......................uuccacagcuuucuugaaUu...........................................................................................................................................................................................   172    1   LT-3
......................uuccacagcuuGcuugaacu...........................................................................................................................................................................................   1      1   JCK-1
......................uuccaAagcuuucuugaacu...........................................................................................................................................................................................   7      1   LCK-3
......................uuccacagcuuucuugaacG...........................................................................................................................................................................................   6      1   LT-3
......................uuccacagcuuucuugaacG...........................................................................................................................................................................................   45     1   LCK-3
......................uuccacagcuuucuugCacu...........................................................................................................................................................................................   10     1   LT-2
......................uCccacagcuuucuugaacu...........................................................................................................................................................................................   3      1   JCK-3
......................uuccCcagcuuucuugaacu...........................................................................................................................................................................................   3      1   LT-1
......................uuccacagcuuucuugaaAu...........................................................................................................................................................................................   6      1   LT-2
......................uuccacagcuuucuuUaacu...........................................................................................................................................................................................   1      1   LT-2
......................uuccUcagcuuucuugaacu...........................................................................................................................................................................................   1      1   JCK-1
......................uuccacCgcuuucuugaacu...........................................................................................................................................................................................   8      1   LT-3
......................uuccacagcCuucuugaacu...........................................................................................................................................................................................   1      1   JCK-3
......................uuccacagcuuucuugaacA...........................................................................................................................................................................................   137    1   LT-2
......................uuccacagcuuucuugaacA...........................................................................................................................................................................................   43     1   JCK-3
......................uNccacagcuuucuugaacu...........................................................................................................................................................................................   2      1   JCK-3
......................Cuccacagcuuucuugaacu...........................................................................................................................................................................................   2      1   LT-1
......................uuccacagcuuucuGgaacu...........................................................................................................................................................................................   1      1   LT-3
......................uNccacagcuuucuugaacuu..........................................................................................................................................................................................   1      1   JT-1
......................uuccacagcuuucuugaacuC..........................................................................................................................................................................................   64     1   JCK-2
......................uuccacagcuuucuugaacuC..........................................................................................................................................................................................   18     1   JT-1
......................uuccacagcuuucuugaaUuu..........................................................................................................................................................................................   135    1   LT-3
......................uuccacagcuuucuuAaacuu..........................................................................................................................................................................................   1      1   JCK-1
......................uuccacagcuuucuugaacAu..........................................................................................................................................................................................   5      1   JCK-2
......................uuccacagcuuuAuugaacuu..........................................................................................................................................................................................   3      1   JCK-3
......................uuccacagcuuucuugCacuu..........................................................................................................................................................................................   2      1   LT-3
......................uuccacGgcuuucuugaacuu..........................................................................................................................................................................................   7      1   JT-2
......................uuccacagcuuNcuugaacuu..........................................................................................................................................................................................   3      1   JCK-1
......................uuccacagcuuuAuugaacuu..........................................................................................................................................................................................   1      1   LT-2
......................uuccacagcuuucCugaacuu..........................................................................................................................................................................................   1      1   LT-3
......................uuccacagcuuuUuugaacuu..........................................................................................................................................................................................   1      1   JCK-2
......................uuccacagcuuucuugaacuu..........................................................................................................................................................................................   1431   0   LT-2
......................uuccacagcuuucuugCacuu..........................................................................................................................................................................................   5      1   JCK-2
......................uuccacagcuuucuugaCcuu..........................................................................................................................................................................................   3      1   JCK-1
......................Cuccacagcuuucuugaacuu..........................................................................................................................................................................................   3      1   JCK-3
......................uuccacagcuuucuugaacNu..........................................................................................................................................................................................   2      1   JCK-1
......................uuccCcagcuuucuugaacuu..........................................................................................................................................................................................   2      1   JCK-1
......................uuccacagcuuucuugaaUuu..........................................................................................................................................................................................   101    1   LT-2
......................uuccacagcuuucuugaacuu..........................................................................................................................................................................................   954    0   LT-1
```

Mature **Star**

uuugcaaguccuggucaugcuu**uuccacagcuuucuugaacu**uucuuucuuucuguuuagucucuuuuuuaagauugaacaccauugaaag**cucaagaaagcugugggaga**auauggcaaauuugggauuuuaauuuuuuuauuuuuguuuuuauuuuuuauuuuuccuucaacuacucuucagcugaugaugauguugcacacagaaagaaagaaagaaagaaagaaagagagaucucagaagcuaaau

uuccacagcuuucuugaacuN	4	1	JCK-1
uuccacagcuuucuugaacuu	2955	0	JCK-3
uuccacagcuuucuugCacuu	2	1	JCK-3
uuccacagcuuucuuAaacuu	1	1	LCK-2
uucGacagcuuucuugaacuu	1	1	JCK-1
uuUcacagcuuucuugaacuu	6	1	JCK-2
uuccacagcuuucuugaacuA	125	1	JCK-2
uuccacagcuCucuugaacuu	1	1	JT-2
uuccacagcuuucuugaacuC	68	1	JCK-3
uuccacagcuuuAuugaacuu	1	1	LCK-3
uuccaNagcuuucuugaacuu	2	1	LCK-1
uuccacagcuuucuugaacuA	134	1	LT-2
uuccacCgcuuucuugaacuu	5	1	JCK-1
uuccacagcuuucuugaacuA	23	1	LCK-2
uCccacagcuuucuugaacuu	1	1	JCK-2
uuccacagcuuucuugaGcuu	4	1	JCK-1
uuccacGgcuuucuugaacuu	1	1	JCK-2
uuccacagcuuucuugaacuu	157	0	LCK-1
uuccacagcuuucuugaacuC	54	1	LT-3
uuccacagcuuucuugaacGu	1	1	JCK-2
uuccacagcuuucuugaacuu	2427	0	LCK-3
uuNcacagcuuucuugaacuu	4	1	LT-2
uuccacagcuuucuuAaacuu	2	1	LCK-3
uCccacagcuuucuugaacuu	1	1	LCK-2
uuccacagcuuucuugaacAu	3	1	JCK-3
uuccacagcuuucuugCacuu	2	1	LT-2
uuccaUagcuuucuugaacuu	1	1	LT-3
uuccaUagcuuucuugaacuu	3	1	JCK-3
uuccacagcuuucuugCacuu	2	1	JCK-1
uuccacaAcuuucuugaacuu	1	1	LCK-3
uuccacagcuuucuugaacuA	97	1	JT-1
uuccacagcuuucuugaaUuu	48	1	LCK-2
uuccacaAcuuucuugaacuu	1	1	LT-1
uuccacagcuuucuugaacuA	2	1	JT-2
uuccacagcuuucCugaacuu	1	1	JCK-2
uucUacagcuuucuugaacuu	4	1	JCK-2
uuUcacagcuuucuugaacuu	1	1	JCK-1
uuccacagcuuucuugaacuA	148	1	LT-3
uuccacagcuuuAuugaacuu	1	1	LT-3
uCccacagcuuucuugaacuu	1	1	LCK-3
uuccacagcuuucuugaacuu	145	0	JT-3
uuccacagcuuucuugaacuA	67	1	JCK-3
uuccacagcuuucuugaaAuu	5	1	LT-2
uuccacagcuuucuugaacuA	66	1	LT-1
uuccCcagcuuucuugaacuu	6	1	JCK-2
uuccacagcuuucuugaacuu	1769	0	LT-3
uuccCcagcuuucuugaacuu	2	1	JCK-3
uuccacagcuuuUuugaacuu	1	1	LT-1
uuccaAagcuuucuugaacuu	1	1	LT-2
uuccacagcuuucuugCacuu	1	1	JT-1
uCccacagcuuucuugaacuu	1	1	LT-2
uucNacagcuuucuugaacuu	1	1	JCK-3
uuccacCgcuuucuugaacuu	1	1	LCK-2
uucNacagcuuucuugaacuu	2	1	JCK-1
uuccacaAcuuucuugaacuu	1	1	LT-2
uuccacagcuuucuugaacuu	191	0	LCK-2
uuccacCgcuuucuugaacuu	3	1	JCK-3
uuccacagcuuucuugaaUuu	112	1	LCK-3
uuccacagcuuucuugaaUuu	11	1	JT-3
uuccacagcuuucCugaacuu	1	1	JCK-1
uuccacagcuuucuugaacCu	1	1	LT-2
uuccacagcuuucuugaacuC	6	1	LCK-1
uuccacagcuuucuugaacuu	2349	0	JCK-1
uuccacagNuuucuugaacuu	1	1	JCK-3
uuccacGgcuuucuugaacuu	1	1	JCK-1
uNccacagcuuucuugaacuu	4	1	JCK-2
uuccacagcuuucuugaCcuu	4	1	LT-2
uuccacagcuuucuCgaacuu	5	1	LT-2
uuccacagcuuucuugaaAuu	1	1	LT-1
uuccacagcuuucuugaacuu	3888	0	JCK-2

Mature Star

uuugcaaguccuggucaugcuuuuccacagcuuucuugaacuuucuuucuuucuguuuagucucuuuuuuaagauugaacaccauugaaagcucaagaaagcugugggagaauauggcaaauuugggauuuuaauuuuuuuauuuuuguuuuuauuuuuuauuuuuccuucaacuacucuucagcugaugaugauguugcacacagaaagaaagaaagaaagaaagaaagagagaucucagaagcuaaau

uuccacagcuuucuugaaUuu	50	1	JCK-1
uuccacagcuuucuugaacGu	1	1	LT-1
uuccacagcuuucuugCacuu	1	1	LCK-3
uuccacagcuuucuugaacuC	44	1	LT-2
uuccacagAuuucuugaacuu	1	1	JCK-1
uNccacagcuuucuugaacuu	1	1	LT-2
uuccCcagcuuucuugaacuu	5	1	LT-2
uuccacagcuuucuugaaUuu	3	1	JT-2
uuccacagcuuucuugaCcuu	3	1	LCK-3
uuccacagcuuucuugaacAu	6	1	LT-2
uucNacagcuuucuugaacuu	1	1	JT-1
uuccacagcuuucuugaCcuu	2	1	JT-1
uuccacagcuuucuugaacuC	53	1	JCK-1
uuUcacagcuuucuugaacuu	2	1	JT-2
uuccacagcuuucuugaaUuu	86	1	LT-1
uuccacaAcuuucuugaacuu	1	1	JCK-3
uuccacagcuuucuugaaUuu	88	1	JCK-2
uuccacagcCuucuugaacuu	2	1	LT-1
uuccacagcuuucuugaacuA	75	1	JCK-1
Cuccacagcuuucuugaacuu	1	1	LT-3
uuUcacagcuuucuugaacuu	2	1	JCK-3
uuccaAagcuuucuugaacuu	5	1	JCK-2
uuccacCgcuuucuugaacuu	3	1	JCK-2
uucUacagcuuucuugaacuu	1	1	JCK-3
uuccacagcuuCcuugaacuu	1	1	LT-2
uuccacCgcuuucuugaacuu	3	1	LT-3
uuccacagcuuucuugaacAu	8	1	LCK-3
uuccacCgcuuucuugaacuu	1	1	JT-1
uuccaAagcuuucuugaacuu	3	1	JCK-3
uuccacagcuuucuugaacuu	97	0	JT-2
uuccacagcuuucuugaacuu	502	0	JT-1
uuccacagcuuucuugaCcuu	1	1	LT-3
uuccacCgcuuucuugaacuu	3	1	LCK-3
uuccacagcuuucuugaacuA	20	1	LCK-1
uuccaAagcuuucuugaacuu	2	1	JCK-1
uNccacagcuuucuugaacuu	1	1	JCK-1
uuccCcagcuuucuugaacuu	1	1	LCK-3
uuccacagcuuucuugCacuu	1	1	LT-1
uuccacagcuuucuugaacAu	6	1	LT-3
uuccacagcuuucuugaacuA	5	1	JT-3
uuUcacagcuuucuugaacuu	1	1	JT-3
uuccCcagcuuucuugaacuu	3	1	LT-3
uuccacagcuuucuugaacuA	240	1	LCK-3
uuccacagcuuucuugaacuC	9	1	LCK-2
uuccacagcuuucuugaacuC	33	1	LT-1
uuccacagcuuucuugaacAu	1	1	LT-1
uNccacagcuuucuugaacuu	6	1	JCK-3
uuccacagcuuucuugaacAu	2	1	JCK-1
uuccacagcuuucuugaaAuu	3	1	LCK-3
uuccacagcuuucuugaacuC	53	1	LCK-3
uuccacagcCuucuugaacuu	1	1	JCK-3
uuccacagcuuucuugaacGu	1	1	LT-3
uuccacagcuuucuugaCcuu	2	1	JCK-3
uuccacGgcuuucuugaacuu	11	1	JT-3
uuccaUagcuuucuugaacuu	1	1	JCK-1
uuccacagcuuucuugaaUuu	29	1	LCK-1
uuccacagcuuucuuAaacuu	1	1	LT-1
uuccacagcuuucuugaaUuu	61	1	JCK-3
uuccacagcuuucuugaCcuu	5	1	JCK-2
uuccacagcuuucuugaacuCu	1	1	LT-1
uuccacagcuuucuCgaacuuu	3	1	LT-3
uuNcacagcuuucuugaacuuu	4	1	LT-2
uuccacagcuuucuugaCcuuu	2	1	JCK-2
uuccGcagcuuucuugaacuuu	1	1	LT-2
uuccacagcuuucuugaaAuuu	2	1	LT-3
uuccacagcCuucuugaacuuu	2	1	JCK-2
uuccacagcuuCcuugaacuuu	1	1	LT-2
uuccacagcuuucuugaacGuu	1	1	LT-3
uuccacagcuuucuugaacuuu	250	0	LCK-1
uuccacagcuuucuugCacuuu	1	1	JCK-1

Mature (uuccacagcuuucuugaacu) **Star** (cucaagaaagcugugggaga)

uuugcaaguccuggucaugcuu**uuccacagcuuucuugaacu**uucuuucuuucuguuuagucucuuuuuuaagauugaacaccauugaaag**cucaagaaagcugugggaga**auauggcaaauuugggauuuuaauuuuuuuauuuuuguuuuauuuuuuauuuuuccuucaacuacucuucagcugaugaugauguugcacacagaaagaaagaaagaaagaaagaaagagagaucucagaagcuaaau

uuccacagcuuucuugaacAuu	16	1	LCK-3
uuccacCgcuuucuugaacuuu	2	1	JCK-2
uCccacagcuuucuugaacuuu	3	1	LT-3
uNccacagcuuucuugaacuuu	2	1	LCK-3
uuccacagcuuucuugaacuuC	118	1	LT-3
uNccacagcuuucuugaacuuu	1	1	JCK-2
Cuccacagcuuucuugaacuuu	1	1	LT-3
uuccacagcuuucuugaacuuu	1068	0	LT-1
uuccaUagcuuucuugaacuuu	1	1	LT-1
uuccacagcuuucuugaacuuA	49	1	JT-1
uuccacagcuuucuugaaUuuu	1	1	JT-3
uuccacagcuuucuugaacuuC	41	1	JT-1
uuccacagcuuucuugaaAuuu	3	1	JCK-2
uuccacagcuuucuugaacCuu	1	1	LT-3
uuccacagcuuucuugaCcuuu	2	1	LT-1
uuccacagcuuucuugaacuuN	1	1	JCK-1
uuccacagcuuucuugaacuuC	253	1	LCK-3
uuccacagcuuucuugaacAuu	36	1	JCK-2
uuccacagcuuucuugaaUuuu	37	1	LT-2
uuccacagcuuucuugaacuuu	246	0	LCK-2
uuccacagcuuucuugaacuAu	13	1	LT-2
uuccacagcCuucuugaacuuu	2	1	LT-2
uuccacagcuuucuugaacGuu	8	1	JCK-2
uuccacagcuuucuugaacuuG	2	1	LT-1
uuccacagcuuucuugaacuuA	121	1	LT-3
uuccacaAcuuucuugaacuuu	1	1	JT-3
uuccacagcuuucuugaacuuC	8	1	JT-2
uuccacagcuuucuugaacuuu	1496	0	JCK-2
uuccacagcuuucuugaacAuu	29	1	LT-3
uuccacagcuuucuugaacuuA	31	1	LCK-2
uuccacagcuuucuugaacuAu	12	1	LT-3
uuccacCgcuuucuugaacuuu	2	1	LCK-2
uuccacagcuuucuugaacuuu	1108	0	JCK-3
uuccacagcuuuAuugaacuuu	4	1	LT-2
uuccacagcuuucuugaaAuuu	2	1	JCK-3
uuccacagcuuucuugaacuuu	70	0	JT-2
uuccacCgcuuucuugaacuuu	1	1	LCK-3
uuccacagcuuucuugaaUuuu	63	1	JCK-2
uuccCcagcuuucuugaacuuu	2	1	JCK-2
uuccacagcuuucuugaacAuu	26	1	LT-2
uuccacagcuuucuugaacuNu	1	1	JCK-1
uucNacagcuuucuugaacuuu	1	1	JCK-1
uCccacagcuuucuugaacuuu	1	1	JCK-3
uuccacagcuuucuugaacuAu	6	1	JCK-3
uuccacagcuGucuugaacuuu	1	1	LT-2
uuccacagcuuucuugaacuCu	1	1	LT-2
uuccacagcuuucuugaacuuC	12	1	JT-3
uuccCcagcuuucuugaacuuu	4	1	LT-3
uuccacCgcuuucuugaacuuu	1	1	JT-1
uuccacagcuuucuugaacAuu	14	1	JCK-1
uuccacCgcuuucuugaacuuu	8	1	LT-2
uuccaAagcuuucuugaacuuu	1	1	JCK-1
uuccacagcuuucuugaacAuu	6	1	JT-2
uuccacagcuuucuugaacuCu	2	1	JCK-1
uuccacagcuuucuugaCcuuu	3	1	LT-2
uuUcacagcuuucuugaacuuu	1	1	JCK-1
uNccacagcuuucuugaacuuu	1	1	JCK-1
uuccacagcuuucuugaacuuA	50	1	JCK-1
uuccacagcuuucuugaacuuA	77	1	JCK-3
uuccacagcuuucuugaacCuu	3	1	JCK-2
uuUcacagcuuucuugaacuuu	1	1	LT-3
uuccacagcuuucuugaacuuA	121	1	LT-2
uuccacaAcuuucuugaacuuu	2	1	LT-2
uuccacagcCuucuugaacuuu	4	1	JCK-3
uuccacagcuuucuugaacCuu	3	1	JCK-3
uuccacagcuuucuugaacGuu	3	1	JCK-1
uuccacagcuuucuugaacuuG	3	1	LT-3
uuccacagcuuucuugaacuuC	55	1	JCK-3
uuccacagcuuucuuUaacuuu	1	1	LT-3
uuccacagcuuucuugaacuAu	11	1	LT-1

```
                     Mature                                                               Star
uugcaaguccuggucaugcuuuuccacagcuuucuugaacuuucuuucuuucuguuuagucucuuuuuuaagauugaacaccauugaaagcucaagaaagcugugggagaauauggcaaauuugggauuuuaauuuuuuuauuuuuguuuuuauuuuuuauuuuuccuucaacuacucuucagcugaugaugauguugcacacagaaagaaagaaagaaagaaagaaagagagaucucagaagcuaaau
.....................uuccacagcuuucuugaacuCu.............................................................................................................................................................................................................    5    1    LCK-3
.....................uuUcacagcuuucuugaacuuu.............................................................................................................................................................................................................    1    1    JCK-2
.....................uuccacagcuuucuugaacCuu.............................................................................................................................................................................................................    1    1    LCK-1
.....................uuccaAagcuuucuugaacuuu.............................................................................................................................................................................................................    1    1    JCK-3
.....................uuccacCgcuuucuugaacuuu.............................................................................................................................................................................................................    3    1    JCK-3
.....................uuccacagcuCucuugaacuuu.............................................................................................................................................................................................................    1    1    LT-1
.....................uuccacagcuuucuugCacuuu.............................................................................................................................................................................................................    1    1    JCK-2
.....................uuccacagcuuucuugaacuCu.............................................................................................................................................................................................................    5    1    JCK-3
.....................uuccCcagcuuucuugaacuuu.............................................................................................................................................................................................................    1    1    LT-1
.....................uuccacagcuuucuugaaUuuu.............................................................................................................................................................................................................    32   1    LT-3
.....................uuUcacagcuuucuugaacuuu.............................................................................................................................................................................................................    7    1    LT-2
.....................uuccacagcuuucuugaacuuu.............................................................................................................................................................................................................    2105 0    LCK-3
.....................uuccacagcuuucuugaacuuu.............................................................................................................................................................................................................    1432 0    LT-2
.....................uuccacagcuuucuugaacAuu.............................................................................................................................................................................................................    12   1    LT-1
.....................uuccCcagcuuucuugaacuuu.............................................................................................................................................................................................................    4    1    LCK-3
.....................uuccacagcuuucuugaacuCu.............................................................................................................................................................................................................    3    1    LT-3
.....................uuccacagcuuucuugaacAuu.............................................................................................................................................................................................................    4    1    JT-3
.....................uuccCcagcuuucuugaacuuu.............................................................................................................................................................................................................    1    1    JCK-1
.....................uuccacagcuuucCugaacuuu.............................................................................................................................................................................................................    1    1    JCK-2
.....................uuccacagcuuucuugaacuuC.............................................................................................................................................................................................................    89   1    JCK-2
.....................uuccacagcuuucuugaaUuuu.............................................................................................................................................................................................................    39   1    LCK-3
.....................uuccacagcuuucuugaacAuu.............................................................................................................................................................................................................    12   1    JCK-3
.....................uuccacagcuuucuugaCcuuu.............................................................................................................................................................................................................    2    1    LT-3
.....................uuGcacagcuuucuugaacuuu.............................................................................................................................................................................................................    2    1    LT-2
.....................uuccacagcuuucuugaacuuG.............................................................................................................................................................................................................    2    1    JCK-2
.....................uuccCcagcuuucuugaacuuu.............................................................................................................................................................................................................    1    1    JCK-3
.....................uuccacagcuuucuugaacuuu.............................................................................................................................................................................................................    1512 0    LT-3
.....................uuccacagcuuucuugaacuuC.............................................................................................................................................................................................................    114  1    LT-2
.....................uuccacagcuuucuugaacuAu.............................................................................................................................................................................................................    16   1    JCK-2
.....................uuccacagcuuucuugaacCuu.............................................................................................................................................................................................................    1    1    JCK-1
.....................uuccacagcuuGcuugaacuuu.............................................................................................................................................................................................................    2    1    JCK-1
.....................uuccacagcuuucuugaaUuuu.............................................................................................................................................................................................................    27   1    JCK-1
.....................uuccaNagcuuucuugaacuuu.............................................................................................................................................................................................................    1    1    LCK-1
.....................uuccacagcuuucuugaacuuN.............................................................................................................................................................................................................    1    1    LT-2
.....................uuccCcagcuuucuugaacuuu.............................................................................................................................................................................................................    3    1    LT-2
.....................uuccacagcuuucuugCacuuu.............................................................................................................................................................................................................    1    1    LCK-3
.....................uuccacagcuuucuugaacuuA.............................................................................................................................................................................................................    17   1    JT-3
.....................uuccacagcuuucuugaacuuG.............................................................................................................................................................................................................    7    1    LT-2
.....................uuccacagcuuucuugCacuuu.............................................................................................................................................................................................................    1    1    LCK-2
.....................uuccacagcuuucuugCacuuu.............................................................................................................................................................................................................    1    1    JCK-3
.....................uuccacagcuuNcuugaacuuu.............................................................................................................................................................................................................    1    1    JT-1
.....................uuccacagcuuucuugCacuuu.............................................................................................................................................................................................................    2    1    LT-1
.....................uuccacagcuuucuugaacuuC.............................................................................................................................................................................................................    73   1    LT-1
.....................uuccacagcuuucuugaacuuA.............................................................................................................................................................................................................    10   1    JT-2
.....................uCccacagcuuucuugaacuuu.............................................................................................................................................................................................................    1    1    LT-1
.....................uuccacagcuuucuugaCcuuu.............................................................................................................................................................................................................    1    1    LCK-3
.....................uuccaAagcuuucuugaacuuu.............................................................................................................................................................................................................    1    1    JCK-2
.....................uuccacCgcuuucuugaacuuu.............................................................................................................................................................................................................    1    1    JCK-1
.....................Cuccacagcuuucuugaacuuu.............................................................................................................................................................................................................    1    1    JCK-1
.....................uuccacagcuuucuugaaUuuu.............................................................................................................................................................................................................    36   1    JCK-3
.....................uuccacagcuuucuugaacuuu.............................................................................................................................................................................................................    377  0    JT-1
.....................uuccacagcuuucuugaaUuuu.............................................................................................................................................................................................................    3    1    JT-2
.....................Cuccacagcuuucuugaacuuu.............................................................................................................................................................................................................    1    1    JCK-3
.....................uuccacagcuuucuugaacuuA.............................................................................................................................................................................................................    128  1    JCK-2
.....................uuccacagcuuucuugaaAuuu.............................................................................................................................................................................................................    2    1    JT-3
.....................Cuccacagcuuucuugaacuuu.............................................................................................................................................................................................................    1    1    JT-1
.....................uuccacagcuuucuugaaUuuu.............................................................................................................................................................................................................    41   1    LT-1
.....................uuccacagcuuucuugaacuAu.............................................................................................................................................................................................................    3    1    JCK-1
.....................uuccacagcuuucuugaaAuuu.............................................................................................................................................................................................................    4    1    JCK-1
.....................uGccacagcuuucuugaacuuu.............................................................................................................................................................................................................    1    1    LCK-3
.....................uuccacagcuuucuugaacuuA.............................................................................................................................................................................................................    62   1    LT-1
.....................uuccacagcuuuAuugaacuuu.............................................................................................................................................................................................................    1    1    LT-1
.....................uuccacagcuuucuugCacuuu.............................................................................................................................................................................................................    1    1    JT-3
.....................uNccacagcuuucuugaacuuu.............................................................................................................................................................................................................    1    1    JCK-3
.....................uuccacagcuuucuugaacuuu.............................................................................................................................................................................................................    899  0    JCK-1
.....................uuccacagcuuucuugaacuuA.............................................................................................................................................................................................................    157  1    LCK-3
.....................uuccacagcuuucuugCacuuu.............................................................................................................................................................................................................    2    1    LT-2
.....................uuccacagcuuNcuugaacuuu.............................................................................................................................................................................................................    3    1    JCK-1
.....................uuccacagcuuucuugaaUuuu.............................................................................................................................................................................................................    7    1    JT-1
.....................uuccacCgcuuucuugaacuuu.............................................................................................................................................................................................................    2    1    LT-1
```

Mature / **Star**

uuugcaaguccuggucaugcuu**uuccacagcuuucuugaacu**uucuuucuuucuguuuagucucuuuuuuaagauugaacaccauugaaag**cucaagaaagcuguggaga**auauggcaaauuugggauuuuaauuuuuuuauuuuuguuuuuauuuuuuauuuuuccuucaacuacucuucagcugaugaugauguugcacacagaaagaaagaaagaaagaaagaaagagagaucucagaagcuaaau

uuccacagcuuucuugaacuuC	65	1	JCK-1
uuccacagcuuuAuugaacuuu	2	1	JCK-1
uuccacagcuGucuugaacuuu	2	1	JCK-1
uuccacagcuuucuugaacuCu	6	1	JCK-2
uuccacagcuuucNugaacuuu	1	1	LCK-1
uuccacCgcuuucuugaacuuu	2	1	LT-3
uuccacagcuuucuugaacuNu	1	1	JT-2
uuccacagcCuucuugaacuuu	2	1	JCK-1
uuccacagcuuucuugaacuuA	21	1	LCK-1
uuccacagcuuucuugaaUuuu	13	1	LCK-2
uuccacagcuuucuugaCcuuu	1	1	LCK-2
uuccacagcuuucuugaacuuu	64	0	JT-3
uuccacagcuuucuugaacuAu	17	1	LCK-3
uucUacagcuuucuugaacuuu	1	1	LCK-3
uuccacagcuuucuugaaUuuu	22	1	LCK-1
uuccacagcuuucuugaacuuuG	13	1	LT-1
uuccacagcuuucuugaacuuuG	4	1	JCK-3
uuccacagcuuucuugaacuuuA	11	1	JT-2
uuccacagcuuucuugaacuuuA	48	1	LCK-2
uuccacagcuuucuugaacuAuc	7	1	LT-2
uuccacagcuuucuugaacuAuc	4	1	LT-1
uuccacagcuuucuugaacuuAc	5	1	JT-3
uuccacagcuuucuugaacuuuG	11	1	LT-3
uuccacagcuuucuugaacAuuc	3	1	LT-3
uuccacagcuuucuugaacuuuc	97	0	LCK-3
uuccacagcuuucuugaacuuuU	294	1	JCK-1
uuccacagcuuucuugaacAuuc	1	1	JCK-1
uuccacagcuuucuugaacuuuU	134	1	LCK-2
uuccacagcuuucuugaacAuuc	10	1	LCK-3
uuNcacagcuuucuugaacuuuc	1	1	LT-2
uuccacagcuuucuugaacuuuU	306	1	LT-1
uuccacagcuuucuugaacuuuA	144	1	JCK-1
uuccacagcuuucuugaacuuuG	8	1	JCK-2
uuccacagcuuucuugaacuAuc	1	1	LT-3
uuccacagcuuucuugaacuuuU	9	1	JT-2
uuccacagcuuucuugaacuuuU	490	1	LCK-3
uuccacagcuuucuugaacuuuc	33	0	JCK-1
uuccacagcuuucuugaacuuAc	1	1	LT-2
uuccacagcuuucuugaacuuCc	1	1	LT-1
uuccacagcuuucuugaacuuuc	28	0	LT-1
uuccacagcuuucuugaacuuuc	61	0	LT-2
uuccacCgcuuucuugaacuuuc	1	1	LT-2
uuccacagcuuucuugaaUuuuc	4	1	LT-1
uuccacagcuuucuugaacuuuU	376	1	LT-2
uuccacagcuuucuugaacuuuU	492	1	JCK-2
uuccacagcuuucuugaacuuuA	177	1	JCK-2
uuccacagcuuucuugaaUuuuc	5	1	JCK-3
uuccacagcuuucuugaacuuuA	230	1	LT-2
uuccacagcuuucuugaacuuuG	9	1	LCK-3
uuccCcagcuuucuugaacuuuc	1	1	LT-2
uuccacagcuuucuugaacuuuU	140	1	LCK-1
uuccacagcuuucuugaacuuuc	40	0	JCK-3
uuccacagcuuucuugaaUuuuc	15	1	LCK-1
uuccacagcuuucuugCacuuuc	1	1	LT-2
uuccacagcuuucuugaacAuuc	2	1	LT-2
uuccacagcuuucuugaacuuAc	1	1	LT-3
uuccacagcuuucuugaacuuuA	119	1	LT-1
uuccacagcuuucuugaacuuuU	18	1	JT-3
uuccacagcuuucuugaacAuuc	1	1	JCK-3
uuccacagcuuucuugCacuuuc	1	1	LCK-3
uuccacagcuuucuugaacuuuA	301	1	LCK-3
uuccacagcuuucuugaacuuuG	4	1	JCK-1
uuccacagcuuucuugaacuuuA	166	1	LT-3
uuccacagcuuucuugaacuuuc	16	0	JT-1
uuccacagcuuucuugaacuuAc	1	1	JT-2
uuccacagcuuucuugaacuuuU	101	1	JT-1
uuccacagcuuucuugaacuuuU	369	1	JCK-3
uuccacagcuuucuugaaUuuuc	3	1	JCK-2
uuccacagcuuucuugaaAuuuc	1	1	LT-3
uuccacagcuuucuugaacuuuA	47	1	JT-1

```
                      Mature                                                                     Star
uuugcaaguccuggucaugcuuuuccacagcuuucuugaacuuucuuucuuucuguuuagucucuuuuuuaagauugaacaccauugaaagcucaagaaagcugugggagaauauggcaaauuugggauuuuaauuuuuuuauuuuuguuuuuauuuuuuauuuuuccuucaacuacucuucagcugaugaugauguugcacacagaaagaaagaaagaaagaaagaaagagagaucucagaagcuaaau
......................uuccacagcuuucuugaaUuuuc............................................................................................................................................................................................................  13   1   LCK-2
......................uuccacGgcuuucuugaacuuuc............................................................................................................................................................................................................  1    1   LT-2
......................uuccacagcuuucuugaacAuuc............................................................................................................................................................................................................  4    1   LT-1
......................uuccacagcuuucuugaacuuuU............................................................................................................................................................................................................  324  1   LT-3
......................uuccacagcuuucuugaaUuuuc............................................................................................................................................................................................................  6    1   LCK-3
......................uuccacagcuuucuugaacuuuc............................................................................................................................................................................................................  3    0   JT-3
......................uuccacagcuuucuugaacuuuc............................................................................................................................................................................................................  47   0   LT-3
......................uuccacagcuuucuugaacuuAc............................................................................................................................................................................................................  1    1   JCK-3
......................uuccacCgcuuucuugaacuuuc............................................................................................................................................................................................................  1    1   LCK-3
......................uuccacagcuuucuugaacuuuc............................................................................................................................................................................................................  50   0   JCK-2
......................uuccacagcuuucuugaacuuuA............................................................................................................................................................................................................  16   1   JT-3
......................uuccacagcuuucuugaacuuuA............................................................................................................................................................................................................  40   1   LCK-1
......................uuccacagcuuucuugaacuuuc............................................................................................................................................................................................................  3    0   JT-2
......................uuccacagcuuucuugaacuuuG............................................................................................................................................................................................................  9    1   LT-2
......................uuccacagcuuucuugaacuuuA............................................................................................................................................................................................................  155  1   JCK-3
......................uuccacagcuuucuugaacuuuUu...........................................................................................................................................................................................................  49   1   JCK-3
......................uuccacagcuuucuugaacuuuAu...........................................................................................................................................................................................................  2    1   LT-2
......................uuccacagcuuucuugaacuAucu...........................................................................................................................................................................................................  1    1   JCK-1
......................uuccacagcuuucuugaacuuuAu...........................................................................................................................................................................................................  5    1   LCK-3
......................uuccacagcuuucuugaacuuAcu...........................................................................................................................................................................................................  2    1   JCK-2
......................uuccacagcuuucuugaacuAucu...........................................................................................................................................................................................................  1    1   JCK-3
......................uuccacagcuuucuugaacuuAcu...........................................................................................................................................................................................................  1    1   LT-3
......................uuccacagcuuucuugaacuAucu...........................................................................................................................................................................................................  4    1   JCK-2
......................uuccacagcuuucuugaacuuucA...........................................................................................................................................................................................................  6    1   JT-1
......................uuccacagcuuucuugaacuuucA...........................................................................................................................................................................................................  1    1   JCK-1
......................uuccacagcuuucuugaacuuucu...........................................................................................................................................................................................................  3    0   LT-1
......................uuccacagcuuucuugaacuuuUu...........................................................................................................................................................................................................  89   1   JCK-2
......................uuccacagcuuucuugaacuuucA...........................................................................................................................................................................................................  1    1   LT-3
......................uuccacagcuuucuugaacuuuUu...........................................................................................................................................................................................................  15   1   LT-2
......................uuccacagcuuucuugaacuuAcu...........................................................................................................................................................................................................  1    1   LT-1
......................uuccacagcuuucuugaacuuuAu...........................................................................................................................................................................................................  2    1   JCK-1
......................uuccacagcuuucuugaacuuuUu...........................................................................................................................................................................................................  13   1   LCK-3
......................uuccacagcuuucuugaacuuucA...........................................................................................................................................................................................................  6    1   LT-2
......................uuccacagcuuucuugaacuuucA...........................................................................................................................................................................................................  2    1   JCK-2
......................uuccacagcuuucuugaacuuucA...........................................................................................................................................................................................................  4    1   JCK-3
......................uuccacagcuuucuugaacuAucu...........................................................................................................................................................................................................  5    1   LCK-3
......................uuccacagcuuucuugaacuuAcu...........................................................................................................................................................................................................  2    1   LT-2
......................uuccacagcuuucuugaacuuuAu...........................................................................................................................................................................................................  4    1   JT-1
......................uuccacagcuuucuugaacuuuAu...........................................................................................................................................................................................................  7    1   JCK-3
......................uuccacagcuuucuugaacuuuAu...........................................................................................................................................................................................................  6    1   LT-3
......................uuccacagcuuucuugaacuuuAu...........................................................................................................................................................................................................  6    1   JCK-2
......................uuccacagcuuucuugaacuuuUu...........................................................................................................................................................................................................  47   1   JCK-1
......................uuccacagcuuucuugaacuuucu...........................................................................................................................................................................................................  2    0   LCK-3
......................uuccacagcuuucuugaacuuuUu...........................................................................................................................................................................................................  13   1   LT-3
......................uuccacagcuuucuugaacuuuAu...........................................................................................................................................................................................................  2    1   LT-1
......................uuccacagcuuucuugaacuuuUu...........................................................................................................................................................................................................  19   1   LT-1
......................uuccacagcuuucuugaacuuucA...........................................................................................................................................................................................................  1    1   LT-1
......................uuccacagcuuucuugaacuuCcu...........................................................................................................................................................................................................  1    1   LCK-3
......................uuccacagcuuucuugaacuuuUuu..........................................................................................................................................................................................................  9    1   JCK-1
......................uuccacagcuuucuugaacuuuAuu..........................................................................................................................................................................................................  8    1   JCK-2
......................uuccacagcuuucuugaacuuuAuu..........................................................................................................................................................................................................  5    1   LT-1
......................uuccacagcuuucuugaacuuuUuu..........................................................................................................................................................................................................  12   1   JCK-2
......................uuccacagcuuucuugaacuuuAuu..........................................................................................................................................................................................................  4    1   LT-2
......................uuccacagcuuucuugaacuuuUuu..........................................................................................................................................................................................................  2    1   LCK-3
......................uuccacagcuuucuugaacuuucuC..........................................................................................................................................................................................................  1    1   LT-3
......................uuccacagcuuucuugaacuuAcuu..........................................................................................................................................................................................................  3    1   JCK-2
......................uuccacagcuuucuugaacuuuUuu..........................................................................................................................................................................................................  1    1   LT-1
......................uuccacagcuuucuugaacuuuAuu..........................................................................................................................................................................................................  12   1   JCK-3
......................uuccacagcuuucuugaacuuuAuu..........................................................................................................................................................................................................  7    1   JCK-1
......................uuccacagcuuucuugaacuuuGuu..........................................................................................................................................................................................................  1    1   LT-3
......................uuccacagcuuucuugaacuuuAuu..........................................................................................................................................................................................................  3    1   LT-3
......................uuccacagcuuucuugaacuuucuA..........................................................................................................................................................................................................  1    1   LT-2
......................uuccacagcuuucuugaacuuuUuu..........................................................................................................................................................................................................  2    1   LT-3
......................uuccacagcuuucuugaacuuuUuu..........................................................................................................................................................................................................  6    1   JCK-3
......................uuccacagcuuucuugaacuuuAuu..........................................................................................................................................................................................................  7    1   LCK-3
......................uuccacagcuuucuugaacuuuUuu..........................................................................................................................................................................................................  5    1   LT-2
......................uuccacagcuuucuugaacuuuAuuu.........................................................................................................................................................................................................  3    1   JCK-2
......................uuccacagcuuucuugaacuuuAuuu.........................................................................................................................................................................................................  5    1   JCK-3
......................uuccacagcuuucuugaacuuuAuuu.........................................................................................................................................................................................................  3    1   JCK-1
......................uuccacagcuuucuugaacuuuUuuu.........................................................................................................................................................................................................  2    1   LCK-3
```

Mature Star

uuugcaaguccuggucaugcuuuuccacagcuuucuugaacuuucuuucuuucuguuuagucucuuuuuuaagauugaacaccauugaaagcucaagaaagcugugggagaauauggcaaauuugggauuuuaauuuuuuuauuuuuguuuuuauuuuuuauuuuuccuucaacuacucuucagcugaugaugauguugcacacagaaagaaagaaagaaagaaagaaagagagaucucagaagcuaaau			
......................uuccacagcuuucuugaacuuuAuuu...	3	1	LCK-3
......................uuccacagcuuucuugaacuuucuuu...	1	0	JCK-1
.......................ucNacagcuuucuugaac...	1	1	LT-2
.......................Cccacagcuuucuugaac...	1	1	LT-1
.......................uccacaUcuuucuugaac...	1	1	LT-3
.......................uccacagcuuucuugaac...	10	0	LT-2
.......................uccacagcuCucuugaac...	1	1	LT-3
.......................uccacagcuuucuugaac...	2	0	JT-2
.......................uccacagcuuucuugaac...	2	0	JCK-2
.......................uccacagcuuucuugaac...	4	0	LT-1
.......................uccacagcuuucuugaac...	13	0	LCK-3
.......................uccacagcuuucuugaac...	6	0	JT-1
.......................uccacagcuuucuugaac...	11	0	LT-3
.......................uccacagcuuucuugaac...	4	0	JT-3
.......................uccacagcuuucuugaac...	2	0	JCK-3
.......................uccacagcuuucuugaacu...	7	0	JCK-3
.......................uccacagcuuucuugaacA...	1	1	LT-3
.......................uccacagcuuucuugaaAu...	3	1	LT-2
.......................uccacagcuuucuugaacu...	7	0	LT-1
.......................uccacagcuuucuugaacu...	12	0	LCK-3
.......................uccacagcuuucuugaacA...	1	1	LT-1
.......................uccacagcuuucuugaacu...	10	0	JCK-2
.......................uccacagcuuucuugaacu...	29	0	LCK-2
.......................uccacagcuuucuugaacu...	2	0	LT-2
.......................uccacagcuuucuugaacu...	18	0	LT-3
.......................uccacagcuuucuugaacu...	2	0	JCK-1
.......................uccacagcuuucuugaacu...	53	0	LCK-1
.......................uccacagcuuucuugaaUu...	1	1	LT-1
.......................uccacagcuuucuugaaUuu...	1	1	LT-3
.......................uccacagcuuucuugaacuu...	6	0	JCK-2
.......................uccacagcuuucuugaacuu...	1	0	JCK-1
.......................uccacagcuuucuugaacuu...	2	0	LT-1
.......................uccacagcuuucuugaacuu...	2	0	LT-3
.......................uccacagcuuucuugaacuu...	5	0	JCK-3
.......................uccacagcuuucuugaacuu...	7	0	LT-2
.......................uccacagcuuucuugaacuu...	9	0	LCK-3
.......................uccacagcuuucuugaacuuu...	4	0	LT-3
.......................uccacagcuuucuugaacuuu...	4	0	LCK-3
.......................uccacagcuuucuugaacuuu...	1	0	JCK-2
.......................Cccacagcuuucuugaacuuu...	1	1	JCK-1
.......................uccacagcuuucuugaacAuu...	4	1	LT-2
.......................uccacagcuuucuugaacuuu...	5	0	LT-2
.......................uccacagcuuucuugaacuuu...	2	0	JCK-1
.......................uccacagcuuucuugaacuuu...	2	0	LT-1
.......................uccacagcuuucuugaacuuC...	1	1	LT-2
.......................uccacagcuuucuugaacuuuU...	1	1	JCK-1
.......................uccacagcuuucuugaacuuuA...	1	1	LT-2
.......................uccacagcuuucuugaacuuuU...	3	1	LT-1
.......................uccacagcuuucuugaacuuuc...	1	0	JCK-2
.......................uccacagcuuucuugaacuuuA...	10	1	LCK-3
.......................uccacagcuuucuugaacuuuU...	1	1	LT-2
.......................uccacagcuuucuugaacuuuc...	1	0	JCK-1
.......................uccacagcuuucuugaacuuuU...	3	1	JCK-2
.......................uccacagcuuucuugaacuuuU...	6	1	LCK-3
.......................uccacagcuuucuugaacuuuc...	2	0	LT-1
.......................uccacagcuuucuugaacuuuU...	2	1	LT-3
.......................uccacagcuuucuugaacuuuU...	2	1	JCK-3
.......................uccacagcuuucuugaacuuuUu...	2	1	LCK-3
........................ccacagcuuucuugaacu...	14	0	JT-2
........................ccacagcuuucuugaCcu...	1	1	JT-1
........................ccacagcuuucuugaaUu...	2	1	LT-1
........................ccacagcCuucuugaacu...	1	1	LT-3
........................ccCcagcuuucuugaacu...	1	1	LT-2
........................ccacagcuuucuugaacG...	1	1	LT-3
........................ccacagcuuucuugaacA...	1	1	LT-3
........................ccacagcuuuUuugaacu...	4	1	LT-3
........................ccacagcuuucuugaaUu...	5	1	LT-3
........................Acacagcuuucuugaacu...	1	1	LT-2
........................ccacagcuuucuugaacu...	51	0	JT-1
........................ccacagcuuucuugaacu...	51	0	JCK-1

Mature **Star**

```
uuugcaaguccuggucaugcuuuuccacagcuuucuugaacuuucuuucuuucuguuuagucucuuuuuuaagauugaacaccauugaaagcucaagaaagcugugggagaauauggcaaauuugggauuuuaauuuuuuuauuuuuguuuuuauuuuuuauuuuuccuucaacuacucuucagcugaugaugauguugcacacagaaagaaagaaagaaagaaagaaagagagaucucagaagcuaaau
........................ccacagcuuucuugaacu................................................................................................................................................................................................................ 301 0 LT-2
........................ccacagcuuucuugaaUu................................................................................................................................................................................................................ 2 1 LCK-3
........................ccacagcuuucuugaCcu................................................................................................................................................................................................................ 1 1 LT-3
........................ccacagcuuucuugaacu................................................................................................................................................................................................................ 36 0 LCK-2
........................ccacagcuuucuugaacC................................................................................................................................................................................................................ 1 1 LT-3
........................ccacagcuuucuugaacu................................................................................................................................................................................................................ 24 0 LCK-1
........................ccacagcuuucuugaacC................................................................................................................................................................................................................ 2 1 JCK-1
........................ccacCgcuuucuugaacu................................................................................................................................................................................................................ 1 1 LT-3
........................ccacagcuuucuugaacu................................................................................................................................................................................................................ 20 0 JT-3
........................ccacagcuuucuugaacA................................................................................................................................................................................................................ 1 1 JCK-2
........................ccacagcuuuAuugaacu................................................................................................................................................................................................................ 1 1 LT-3
........................ccacagcuuucuugaacA................................................................................................................................................................................................................ 2 1 LT-2
........................ccacagcuuucuugaacu................................................................................................................................................................................................................ 104 0 LT-1
........................ccacagcuuucuugaacu................................................................................................................................................................................................................ 69 0 JCK-3
........................ccacagcuuucuugaacu................................................................................................................................................................................................................ 318 0 LCK-3
........................ccacagcuuucuugCacu................................................................................................................................................................................................................ 2 1 LT-3
........................ccacagcuuucuugaacu................................................................................................................................................................................................................ 89 0 JCK-2
........................ccacagcuuucuugaacu................................................................................................................................................................................................................ 342 0 LT-3
........................ccacagcuuucuugaacA................................................................................................................................................................................................................ 1 1 JCK-1
........................ccacagcuuucuugaCcu................................................................................................................................................................................................................ 2 1 LCK-3
........................ccacCgcuuucuugaacuu............................................................................................................................................................................................................... 1 1 JT-1
........................ccacagcuuucuugaaAuu............................................................................................................................................................................................................... 1 1 JCK-2
........................ccacagcuuucuugaacuu............................................................................................................................................................................................................... 34 0 JT-2
........................ccacagcuuucuugaacAu............................................................................................................................................................................................................... 1 1 JCK-1
........................ccacagcuuucuugaacuC............................................................................................................................................................................................................... 2 1 JCK-1
........................ccacagcuuucuugaacuC............................................................................................................................................................................................................... 10 1 JT-1
........................ccacagcuuucuugaacCu............................................................................................................................................................................................................... 1 1 LCK-3
........................ccacagcuuucuugaaAuu............................................................................................................................................................................................................... 3 1 LT-3
........................cNacagcuuucuugaacuu............................................................................................................................................................................................................... 1 1 JCK-2
........................ccacagcuuucuugaacuu............................................................................................................................................................................................................... 251 0 LT-3
........................ccacagcuuucuugaaUuu............................................................................................................................................................................................................... 4 1 JCK-3
........................ccacagcuuucuugaacuC............................................................................................................................................................................................................... 5 1 LT-1
........................ccaNagcuuucuugaacuu............................................................................................................................................................................................................... 1 1 JCK-1
........................ccacagcuuucuugaCcuu............................................................................................................................................................................................................... 1 1 JT-2
........................ccacagcuuucuugaaUuu............................................................................................................................................................................................................... 5 1 JCK-2
........................ccacagcuuucuugaacuu............................................................................................................................................................................................................... 168 0 JCK-1
........................ccacagcuuucuugaacuC............................................................................................................................................................................................................... 4 1 LT-3
........................ccacagcuuucuugaacuA............................................................................................................................................................................................................... 41 1 LCK-3
........................ccacagcuuucuugaacuu............................................................................................................................................................................................................... 66 0 LT-1
........................ccacagcuuucuugaacuu............................................................................................................................................................................................................... 252 0 JCK-2
........................ccacagcuuucuugaacuu............................................................................................................................................................................................................... 68 0 JT-1
........................ccacagcuuucuugaaUuu............................................................................................................................................................................................................... 6 1 LCK-3
........................ccacagcuuucuugaacuA............................................................................................................................................................................................................... 6 1 LT-1
........................ccacagcuuucuugaacuA............................................................................................................................................................................................................... 8 1 JCK-3
........................cNacagcuuucuugaacuu............................................................................................................................................................................................................... 1 1 JCK-3
........................ccacagcuuucuugaacuA............................................................................................................................................................................................................... 9 1 LT-3
........................ccacagcuuucuugaacuC............................................................................................................................................................................................................... 3 1 LT-2
........................ccacCgcuuucuugaacuu............................................................................................................................................................................................................... 1 1 JCK-2
........................ccacagcuuucuugaaUuu............................................................................................................................................................................................................... 3 1 JCK-1
........................ccacCgcuuucuugaacuu............................................................................................................................................................................................................... 2 1 LCK-3
........................ccacagcuuucuugaacuA............................................................................................................................................................................................................... 10 1 JT-1
........................ccacagcuuucuugaaUuu............................................................................................................................................................................................................... 7 1 LT-3
........................ccacagcuuucuugaacuu............................................................................................................................................................................................................... 349 0 LCK-3
........................ccacagcuuucuugaCcuu............................................................................................................................................................................................................... 1 1 LCK-3
........................ccacagcuuucuugaacuu............................................................................................................................................................................................................... 42 0 JT-3
........................ccacagcuuucuugaCcuu............................................................................................................................................................................................................... 1 1 LT-2
........................cNacagcuuucuugaacuu............................................................................................................................................................................................................... 1 1 JT-1
........................ccacagcuuucuugaacuC............................................................................................................................................................................................................... 4 1 JCK-3
........................ccacagcuuucuugaacuA............................................................................................................................................................................................................... 11 1 JCK-1
........................ccacagcuuucuugaacuA............................................................................................................................................................................................................... 17 1 JCK-2
........................ccacagcuuucuugaacuA............................................................................................................................................................................................................... 19 1 LT-2
........................ccacagcuuucuugaacuu............................................................................................................................................................................................................... 246 0 LT-2
........................ccacagcuuucuugaaUuu............................................................................................................................................................................................................... 3 1 LT-1
........................ccacagcuuucuugCacuu............................................................................................................................................................................................................... 2 1 LT-2
........................ccacagcuuucuugaacuA............................................................................................................................................................................................................... 5 1 JT-3
........................ccacagcuuucuugCacuu............................................................................................................................................................................................................... 2 1 JCK-3
........................ccacagcuuucuugaacuA............................................................................................................................................................................................................... 3 1 JT-2
........................ccacagcuuucuugCacuu............................................................................................................................................................................................................... 1 1 JCK-2
........................ccacagcCuucuugaacuu............................................................................................................................................................................................................... 3 1 LCK-3
........................ccCcagcuuucuugaacuu............................................................................................................................................................................................................... 1 1 LT-3
```

```
                      Mature                                                                               Star
uuugcaaguccuggucaugcuuuuccacagcuuucuugaacuuucuuucuuucuguuuagucucuuuuuuaagauugaacaccauugaaagcucaagaaagcugugggagaauauggcaaauuugggauuuuaauuuuuuuauuuuuguuuuuauuuuuuauuuuuccuucaacuacucuucagcugaugaugauguugcacacagaaagaaagaaagaaagaaagaaagagagaucucagaagcuaaau
........................ccacagcuuucNugaacuu....................................................................................................................................................................................................................  1    1  JCK-1
........................ccaAagcuuucuugaacuu....................................................................................................................................................................................................................  1    1  JCK-1
........................ccacagcuuucuugaacuC....................................................................................................................................................................................................................  12   1  JCK-2
........................ccacagcuuucuugaaUuu....................................................................................................................................................................................................................  9    1  LT-2
........................ccacagcuuucuugaacuu....................................................................................................................................................................................................................  180  0  JCK-3
........................ccacagcuuucuugaacuuG...................................................................................................................................................................................................................  7    1  JCK-1
........................ccacagcuuucuugaacuCu...................................................................................................................................................................................................................  8    1  LT-3
........................ccacagcuuucuugaacuCu...................................................................................................................................................................................................................  17   1  JCK-2
........................ccacagcuuucuugaacuCu...................................................................................................................................................................................................................  4    1  JCK-1
........................ccacagcuuucuugaacAuu...................................................................................................................................................................................................................  2    1  JCK-1
........................ccacagcuuucuugaacuuu...................................................................................................................................................................................................................  43   0  JT-1
........................ccacagcuuucuugaacuuG...................................................................................................................................................................................................................  1    1  LT-3
........................ccacagcuuucuugaacuuA...................................................................................................................................................................................................................  53   1  JCK-3
........................ccacagcuuucuugaaUuuu...................................................................................................................................................................................................................  3    1  JCK-3
........................ccacagcuuucuugaacAuu...................................................................................................................................................................................................................  3    1  LT-2
........................ccacagcuuucuugaacuuC...................................................................................................................................................................................................................  18   1  JCK-3
........................ccNcagcuuucuugaacuuu...................................................................................................................................................................................................................  1    1  LCK-1
........................ccacagcuuucuugaacuuC...................................................................................................................................................................................................................  5    1  LCK-3
........................ccacagcuuucuugaacuuA...................................................................................................................................................................................................................  8    1  JT-3
........................ccacaNcuuucuugaacuuu...................................................................................................................................................................................................................  1    1  LCK-1
........................ccacagcuuucuugaacuuu...................................................................................................................................................................................................................  485  0  LT-2
........................ccacagcuuucuugaacuuA...................................................................................................................................................................................................................  51   1  LT-2
........................ccacagcuuucuugaCcuuu...................................................................................................................................................................................................................  1    1  JCK-3
........................ccacagcuuucuugaacuuu...................................................................................................................................................................................................................  71   0  LCK-2
........................ccacagcuuucuugCacuuu...................................................................................................................................................................................................................  1    1  JCK-2
........................ccacagcuuucuugaacAuu...................................................................................................................................................................................................................  5    1  LCK-3
........................ccacagcuuucuugaacuuG...................................................................................................................................................................................................................  1    1  JCK-3
........................ccacCgcuuucuugaacuuu...................................................................................................................................................................................................................  1    1  JT-1
........................ccacagcuuucuugaacuCu...................................................................................................................................................................................................................  7    1  JCK-3
........................ccacagGuuucuugaacuuu...................................................................................................................................................................................................................  1    1  JT-3
........................ccacCgcuuucuugaacuuu...................................................................................................................................................................................................................  3    1  JCK-3
........................ccacCgcuuucuugaacuuu...................................................................................................................................................................................................................  1    1  JCK-1
........................ccacagcuuucuugaacuuA...................................................................................................................................................................................................................  10   1  LT-1
........................ccacagcuuucuugNacuuu...................................................................................................................................................................................................................  1    1  LCK-1
........................ccacagcuuucuugaacuAu...................................................................................................................................................................................................................  9    1  LCK-3
........................Ucacagcuuucuugaacuuu...................................................................................................................................................................................................................  1    1  JCK-1
........................ccacagcuuucuugaacuuA...................................................................................................................................................................................................................  48   1  LT-3
........................ccacagcuuucuugaacuAu...................................................................................................................................................................................................................  5    1  JCK-3
........................ccacagcuuucuugaacuuu...................................................................................................................................................................................................................  77   0  LCK-1
........................ccacagcuuucuugaacuuA...................................................................................................................................................................................................................  69   1  JCK-2
........................ccacagcuuCcuugaacuuu...................................................................................................................................................................................................................  1    1  LT-3
........................ccacagcuuucuugaaUuuu...................................................................................................................................................................................................................  7    1  LT-3
........................ccacagcuuucuugCacuuu...................................................................................................................................................................................................................  1    1  LT-2
........................ccacagcuuucuugaacGuu...................................................................................................................................................................................................................  1    1  JCK-1
........................ccacagcuuucuuAaacuuu...................................................................................................................................................................................................................  1    1  LT-2
........................ccacagcuuucuugaacuuA...................................................................................................................................................................................................................  40   1  LCK-3
........................ccacagcCuucuugaacuuu...................................................................................................................................................................................................................  1    1  JCK-3
........................ccacagcuuucuugaaUuuu...................................................................................................................................................................................................................  13   1  LCK-3
........................ccacagcuuucuugaacuuG...................................................................................................................................................................................................................  2    1  JCK-2
........................ccacagcuuucuugaacuuu...................................................................................................................................................................................................................  169  0  LT-1
........................ccacagcuuucuugaacuCu...................................................................................................................................................................................................................  4    1  LT-2
........................ccacagcuuucuugaacuuC...................................................................................................................................................................................................................  15   1  JCK-1
........................ccacCgcuuucuugaacuuu...................................................................................................................................................................................................................  2    1  LT-2
........................Ucacagcuuucuugaacuuu...................................................................................................................................................................................................................  4    1  JCK-2
........................ccacagcuuucuugaacuuu...................................................................................................................................................................................................................  21   0  JT-2
........................ccacagcuuucuugaacuuu...................................................................................................................................................................................................................  349  0  JCK-3
........................ccacagcuuucuugaGcuuu...................................................................................................................................................................................................................  1    1  LCK-2
........................ccacagcuuucuugaacuuC...................................................................................................................................................................................................................  14   1  LT-2
........................ccacagcuuucuugaacuAu...................................................................................................................................................................................................................  16   1  LT-2
........................ccacagcuuucuugaacuAu...................................................................................................................................................................................................................  6    1  JCK-1
........................ccacagcuuucuugaacuuC...................................................................................................................................................................................................................  20   1  LT-3
........................ccacagcuuucuugaacuuA...................................................................................................................................................................................................................  11   1  JT-2
........................ccacagcuuucuugaacAuu...................................................................................................................................................................................................................  2    1  LT-3
........................ccacagcuuucuugaaUuuu...................................................................................................................................................................................................................  27   1  LCK-1
........................ccacagcuuucuugaaUuuu...................................................................................................................................................................................................................  18   1  LCK-2
........................ccacagcuuucuugaacuuu...................................................................................................................................................................................................................  439  0  LT-3
........................ccacagcuuucuugaacAuu...................................................................................................................................................................................................................  1    1  JCK-2
........................ccacagcuuucuugaacuCu...................................................................................................................................................................................................................  4    1  LT-1
........................ccacagcuuucuugaacuAu...................................................................................................................................................................................................................  9    1  LT-3
........................ccaAagcuuucuugaacuuu...................................................................................................................................................................................................................  1    1  LCK-1
```

Mature Star

```
uuugcaaguccuggucaugcuuuuccacagcuuucuugaacuuucuuucuuucuguuuagucucuuuuuuaagauugaacaccauugaaagcucaagaaagcugugggagaauauggcaaauuugggauuuuaauuuuuuuauuuuuguuuuuauuuuuuauuuuuccuucaacuacucuucagcugaugaugauguugcacacagaaagaaagaaagaaagaaagaaagagagaucucagaagcuaaau
........................ccacaAcuuucuugaacuuu..............................................................................................................................................................................   1     1   JCK-3
........................ccacagcuuucuugaacuAu..............................................................................................................................................................................   9     1   JCK-2
........................ccacagcuuucuugaacAuu..............................................................................................................................................................................   2     1   LT-1
........................ccacagcuuucuugaacuuu..............................................................................................................................................................................   495   0   JCK-2
........................ccacagcuuucuugaacuuA..............................................................................................................................................................................   41    1   JCK-1
........................cNacagcuuucuugaacuuu..............................................................................................................................................................................   1     1   JCK-1
........................ccacagcuuucuugaacuuC..............................................................................................................................................................................   4     1   LT-1
........................ccacagcuuucuugaacuuu..............................................................................................................................................................................   334   0   JCK-1
........................ccacagcuuucuugCacuuu..............................................................................................................................................................................   1     1   LCK-3
........................ccacagcuuucuugaacuuC..............................................................................................................................................................................   24    1   JCK-2
........................ccacagcuuucuugaaUuuu..............................................................................................................................................................................   4     1   JCK-1
........................ccacagcuuucuugaacuuu..............................................................................................................................................................................   506   0   LCK-3
........................ccacagcuuucuugaacuuG..............................................................................................................................................................................   1     1   LT-1
........................ccacagcuuucuugaacuuu..............................................................................................................................................................................   31    0   JT-3
........................ccacagcuuucuugaaAuuu..............................................................................................................................................................................   5     1   LCK-3
........................ccacagcuuucuugaacuuuU.............................................................................................................................................................................   2     1   JT-2
........................ccacagcuuucuugaacuuuA.............................................................................................................................................................................   146   1   LT-2
........................ccacagcuuucuugaacuuuA.............................................................................................................................................................................   109   1   LCK-3
........................ccacagcuuucuugaacuuuA.............................................................................................................................................................................   33    1   LT-1
........................ccacagcuuucuugaacuuuU.............................................................................................................................................................................   184   1   JCK-2
........................ccacagcuuucuugaCcuuuc.............................................................................................................................................................................   1     1   LT-1
........................ccacagcuuucuugaacuuuA.............................................................................................................................................................................   7     1   JT-1
........................ccacagcuuucuugaacuuAc.............................................................................................................................................................................   5     1   LCK-3
........................ccacagcuuucuugaacuuuG.............................................................................................................................................................................   10    1   JCK-3
........................ccacagcuuucuugaacuuuU.............................................................................................................................................................................   246   1   LT-2
........................ccacagcuuucuugaacuuuU.............................................................................................................................................................................   42    1   JT-1
........................ccacagcuuucuugaacuuuc.............................................................................................................................................................................   3     0   LT-1
........................ccacagcuuucuugaacuuuA.............................................................................................................................................................................   178   1   JCK-2
........................ccacagcuuucuugaacuuuU.............................................................................................................................................................................   138   1   JCK-1
........................ccacagcuuucuugaacuuuc.............................................................................................................................................................................   29    0   LCK-3
........................ccacagcuuucuugaacuuuG.............................................................................................................................................................................   6     1   LT-1
........................ccacagcuuucuugaacuAuc.............................................................................................................................................................................   7     1   LCK-3
........................ccacagcuuucuugaaUuuuc.............................................................................................................................................................................   1     1   LT-2
........................ccacagcuuucuugaacuuuG.............................................................................................................................................................................   17    1   LCK-3
........................ccacagcuuucuugaacuuuU.............................................................................................................................................................................   13    1   JT-3
........................ccacagcuuucuugaacuuCc.............................................................................................................................................................................   1     1   LCK-3
........................ccacagcuuucuugaacuAuc.............................................................................................................................................................................   1     1   LT-3
........................ccacagcuuucuugaacuuuU.............................................................................................................................................................................   87    1   LT-1
........................ccacagcuuucuugaacuuuA.............................................................................................................................................................................   90    1   JCK-3
........................ccacagcuuucuugaacuuCc.............................................................................................................................................................................   1     1   JCK-1
........................ccacagcuuucuugaacuuAc.............................................................................................................................................................................   1     1   JCK-2
........................ccacagcuuucuugaacuuuA.............................................................................................................................................................................   150   1   JCK-1
........................ccacagcuuucuugaacuuCc.............................................................................................................................................................................   1     1   LT-3
........................ccacagcuuucuugaacuuuG.............................................................................................................................................................................   8     1   JCK-2
........................ccacagcuuucuugaacuuuc.............................................................................................................................................................................   27    0   LCK-1
........................ccacagcuuucuugaacuuuU.............................................................................................................................................................................   153   1   JCK-3
........................ccacagcuuucuugaacuuuU.............................................................................................................................................................................   64    1   LCK-2
........................ccacagcuuucuugaacuuuU.............................................................................................................................................................................   223   1   LT-3
........................ccacagcuuucuugaacuuuc.............................................................................................................................................................................   16    0   LT-2
........................ccacagcuuucuugaacuuuU.............................................................................................................................................................................   349   1   LCK-3
........................ccacagcuuucuugaacuuuG.............................................................................................................................................................................   14    1   LT-2
........................ccacagcuuucuugaacuuuG.............................................................................................................................................................................   6     1   LT-3
........................ccacagcuuucuugaacuuuc.............................................................................................................................................................................   30    0   LCK-2
........................ccacagcuuucuugaacuuuA.............................................................................................................................................................................   82    1   LT-3
........................ccacagcuuucuugaacuuAc.............................................................................................................................................................................   1     1   LT-2
........................ccacagcuuucuugaacuuuc.............................................................................................................................................................................   4     0   JCK-1
........................ccacagcuuucuugaacuuAc.............................................................................................................................................................................   3     1   LT-3
........................ccacagcuuucuugaacuuuc.............................................................................................................................................................................   8     0   JCK-2
........................ccacagcuuucuugaacuuuU.............................................................................................................................................................................   52    1   LCK-1
........................ccacagcuuucuugaacuuuc.............................................................................................................................................................................   8     0   LT-3
........................ccacagcuuucuugaacuuuG.............................................................................................................................................................................   9     1   JCK-1
........................ccacagcuuucuugaacuuuc.............................................................................................................................................................................   9     0   JCK-3
........................ccacagcuuucuugaacuuuGu............................................................................................................................................................................   3     1   LT-1
........................ccacagcuuucuugaacuuucA............................................................................................................................................................................   1     1   JCK-1
........................ccacagcuuucuugaacuuucA............................................................................................................................................................................   5     1   JCK-2
........................ccacagcuuucuugaacuuucA............................................................................................................................................................................   3     1   LT-3
........................ccacagcuuucuugaacuuuUu............................................................................................................................................................................   25    1   LT-3
........................ccacagcuuucuugaacuuuUu............................................................................................................................................................................   38    1   JCK-3
........................ccacagcuuucuugaacuuuGu............................................................................................................................................................................   5     1   LCK-3
........................ccacagcuuucuugaacuuucG............................................................................................................................................................................   1     1   JCK-3
```

Mature Star

```
uuugcaaguccuggucaugcuuuuccacagcuuucuugaacuuucuuucuuucuguuuagucucuuuuuuaagauugaacaccauugaaagcucaagaaagcugugggagaauauggcaaauuugggauuuuaauuuuuuuauuuuuguuuuuauuuuuuauuuuuccuucaacuacucuucagcugaugaugauguugcacacagaaagaaagaaagaaagaaagaaagagagaucucagaagcuaaau
........................ccacagcuuucuugaacuuAcu........................................................................................................................................................................................................   4   1   JCK-2
........................ccacagcuuucuugaacuuucu........................................................................................................................................................................................................   4   0   JCK-3
........................ccacagcuuucuugaacuuuUu........................................................................................................................................................................................................   4   1   JT-3
........................ccacagcuuucuugaacuuucA........................................................................................................................................................................................................   3   1   JCK-3
........................ccacagcuuucuugaacuuucu........................................................................................................................................................................................................   4   0   JCK-2
........................ccacagcuuucuugaacuuucu........................................................................................................................................................................................................   1   0   LT-1
........................ccacagcuuucuugaacuuAcu........................................................................................................................................................................................................   1   1   JCK-1
........................ccacagcuuucuugaacuuucC........................................................................................................................................................................................................   2   1   LT-3
........................ccacagcuuucuugaacuuucu........................................................................................................................................................................................................   3   0   LT-3
........................ccacagcuuucuugaacuuuUu........................................................................................................................................................................................................   39  1   LT-2
........................ccacagcuuucuugaacuuAcu........................................................................................................................................................................................................   1   1   LT-1
........................ccacagcuuucuugaacuuAcu........................................................................................................................................................................................................   4   1   JCK-3
........................ccacagcuuucuugaacuuucA........................................................................................................................................................................................................   4   1   LT-2
........................ccacagcuuucuugaacuuuAu........................................................................................................................................................................................................   16  1   JCK-3
........................ccacagcuuucuugaacuuuAu........................................................................................................................................................................................................   19  1   JCK-1
........................ccacagcuuucuugaacuuuUu........................................................................................................................................................................................................   54  1   JCK-2
........................ccacagcuuucuugaacuuuAu........................................................................................................................................................................................................   23  1   LCK-3
........................ccacagcuuucuugaacuuuAu........................................................................................................................................................................................................   4   1   LT-1
........................ccacagcuuucuugaacuAucu........................................................................................................................................................................................................   2   1   LCK-3
........................ccacagcuuucuugaacuuAcu........................................................................................................................................................................................................   23  1   LCK-3
........................ccacagcuuucuugaacuuuAu........................................................................................................................................................................................................   1   1   JT-3
........................ccacagcuuucuugaacuuuAu........................................................................................................................................................................................................   16  1   LT-2
........................ccacagcuuucuugaacuuuUu........................................................................................................................................................................................................   29  1   JCK-1
........................ccacagcuuucuugaacuuuUu........................................................................................................................................................................................................   22  1   LCK-3
........................ccacagcuuucuugaacuuuAu........................................................................................................................................................................................................   23  1   JCK-2
........................ccacagcuuucuugaacuuAcu........................................................................................................................................................................................................   3   1   LT-2
........................ccacagcuuucuugaacuuucu........................................................................................................................................................................................................   1   0   JCK-1
........................ccacagcuuucuugaacuuuUu........................................................................................................................................................................................................   7   1   LT-1
........................ccacagcuuucuugaacuuucG........................................................................................................................................................................................................   5   1   JCK-2
........................ccacagcuuucuugaacuuuAu........................................................................................................................................................................................................   13  1   LT-3
........................ccacagcuuucuugaacuuuUu........................................................................................................................................................................................................   2   1   JT-2
........................ccacagcuuucuugaacuuAcu........................................................................................................................................................................................................   2   1   LT-3
........................ccacagcuuucuugaacuuuAuu.......................................................................................................................................................................................................   14  1   JCK-1
........................ccacagcuuucuugaacuuuUuu.......................................................................................................................................................................................................   5   1   LT-2
........................ccacagcuuucuugaacuuucuu.......................................................................................................................................................................................................   1   0   JCK-3
........................ccacagcuuucuugaacuuucCu.......................................................................................................................................................................................................   1   1   JCK-2
........................ccacagcuuucuugaacuuucAu.......................................................................................................................................................................................................   1   1   JCK-3
........................ccacagcuuucuugaacuuuAuu.......................................................................................................................................................................................................   13  1   JCK-3
........................ccacagcuuucuugaacuuucAu.......................................................................................................................................................................................................   1   1   JCK-2
........................ccacagcuuucuugaacuuuAuu.......................................................................................................................................................................................................   4   1   LT-3
........................ccacagcuuucuugaacuuucuA.......................................................................................................................................................................................................   1   1   LT-2
........................ccacagcuuucuugaacuuuGuu.......................................................................................................................................................................................................   1   1   JCK-2
........................ccacagcuuucuugaacuuuAuu.......................................................................................................................................................................................................   4   1   JT-3
........................ccacagcuuucuugaacuuuAuu.......................................................................................................................................................................................................   25  1   LCK-3
........................ccacagcuuucuugaacuuuAuu.......................................................................................................................................................................................................   1   1   JT-2
........................ccacagcuuucuugaacuuuUuu.......................................................................................................................................................................................................   14  1   LCK-3
........................ccacagcuuucuugaacuuuUuu.......................................................................................................................................................................................................   4   1   JCK-1
........................ccacagcuuucuugaacuuuUuu.......................................................................................................................................................................................................   9   1   JCK-3
........................ccacagcuuucuugaacuuuAuu.......................................................................................................................................................................................................   13  1   LT-2
........................ccacagcuuucuugaacuuuAuu.......................................................................................................................................................................................................   21  1   JCK-2
........................ccacagcuuucuugaacuuuUuu.......................................................................................................................................................................................................   4   1   LT-3
........................ccacagcuuucuugaacuuuUuu.......................................................................................................................................................................................................   11  1   JCK-2
........................ccacagcuuucuugaacuuuUuu.......................................................................................................................................................................................................   5   1   LT-1
........................ccacagcuuucuugaacuuuAuu.......................................................................................................................................................................................................   3   1   LT-1
........................ccacagcuuucuugaacuuucuA.......................................................................................................................................................................................................   2   1   LCK-3
........................ccacagcuuucuugaacuuuAuuu......................................................................................................................................................................................................   2   1   JCK-2
........................ccacagcuuucuugaacuuuAuuu......................................................................................................................................................................................................   1   1   LT-1
........................ccacagcuuucuugaacuuucuCu......................................................................................................................................................................................................   1   1   JCK-2
........................ccacagcuuucuugaacuuuUuuu......................................................................................................................................................................................................   2   1   JCK-2
........................ccacagcuuucuugaacuuuAuuu......................................................................................................................................................................................................   1   1   LT-2
........................ccacagcuuucuugaacuuuAuuu......................................................................................................................................................................................................   1   1   LT-3
........................ccacagcuuucuugaacuuuAuuu......................................................................................................................................................................................................   3   1   JCK-1
........................ccacagcuuucuugaacuuuUuuu......................................................................................................................................................................................................   3   1   LT-3
........................ccacagcuuucuugaacuuuUuuu......................................................................................................................................................................................................   1   1   LT-2
........................ccacagcuuucuugaacuuucAuu......................................................................................................................................................................................................   1   1   JCK-2
........................ccacagcuuucuugaacuuuUuuu......................................................................................................................................................................................................   3   1   JCK-3
........................ccacagcuuucuugaacuuuAuuu......................................................................................................................................................................................................   1   1   JCK-3
........................ccacagcuuucuugaacuuuUuuu......................................................................................................................................................................................................   2   1   JCK-1
.........................cacagcuuucuugaacuu...........................................................................................................................................................................................................   5   0   LCK-3
.........................cacagcuuucuugaacuu...........................................................................................................................................................................................................   3   0   LT-2
```

```
                         Mature                                                                   Star
uuugcaaguccuggucaugcuuuuccacagcuuucuugaacuuucuuucuuucuguuuagucucuuuuuuaagauugaacaccauugaaagcucaagaaagcugugggagaauauggcaaauuugggauuuuaauuuuuuuauuuuuguuuuuauuuuuuauuuuuccuucaacuacucuucagcugaugaugauguugcacacagaaagaaagaaagaaagaaagaaagagagaucucagaagcuaaau
.........................cacagcuuucuugaacuu...............................................................................................................................................................................................................   3   0   JCK-3
.........................cacagcuuucuugaacuu...............................................................................................................................................................................................................   1   0   LT-3
.........................cCcagcuuucuugaacuu...............................................................................................................................................................................................................   1   1   LCK-3
.........................cacagcuuucuugaacuuu..............................................................................................................................................................................................................   2   0   JCK-3
.........................cacagcuuucuugaacuuu..............................................................................................................................................................................................................   6   0   LT-3
.........................cacagcuuucuugaacuuA..............................................................................................................................................................................................................   1   1   LT-3
.........................cacagcuuucuugaacuuA..............................................................................................................................................................................................................   2   1   JCK-3
.........................cacagcuuucuugaacuuu..............................................................................................................................................................................................................   1   0   JT-2
.........................cacagcuuucuugaacuuu..............................................................................................................................................................................................................   10  0   JT-3
.........................cacagcuuucuugaacuuu..............................................................................................................................................................................................................   2   0   LT-1
.........................cacagcuuucuugaacuAu..............................................................................................................................................................................................................   2   1   LT-2
.........................cacagcuuucuugaacuuA..............................................................................................................................................................................................................   1   1   JCK-1
.........................cacagcuuucuugaacuuu..............................................................................................................................................................................................................   8   0   JCK-2
.........................cacagcuuucuugaacuuu..............................................................................................................................................................................................................   1   0   JCK-1
.........................cacagcuuucuugaacuuu..............................................................................................................................................................................................................   4   0   LT-2
.........................cacagcuuucuugaacuuuU.............................................................................................................................................................................................................   3   1   JCK-1
.........................cacagcuuucuugaacuuuA.............................................................................................................................................................................................................   1   1   LT-3
.........................cacagcuuucuugaacuuuU.............................................................................................................................................................................................................   1   1   LT-1
.........................cacagcuuucuugaacuuuU.............................................................................................................................................................................................................   5   1   JCK-3
.........................cacagcuuucuugaacuuuA.............................................................................................................................................................................................................   1   1   JCK-3
.........................cacagcuuucuugaacuuuU.............................................................................................................................................................................................................   2   1   LT-2
.........................cacagcuuucuugaacuuuU.............................................................................................................................................................................................................   5   1   JCK-2
.........................cacagcuuucuugaacuuuA.............................................................................................................................................................................................................   4   1   LCK-3
.........................cacagcuuucuugaacuuuU.............................................................................................................................................................................................................   2   1   LT-3
.........................cacagcuuucuugaacuuuU.............................................................................................................................................................................................................   5   1   LCK-3
.........................cacagcuuucuugaacuuucu............................................................................................................................................................................................................   2   0   JCK-2
.........................cacagcuuucuugaacuuucu............................................................................................................................................................................................................   3   0   LT-1
.........................cacagcuuucuugaacuuucu............................................................................................................................................................................................................   9   0   JCK-3
.........................cacagcuuucuugaacuuucu............................................................................................................................................................................................................   2   0   JCK-1
.........................cacagcuuucuugaacuuuUuu...........................................................................................................................................................................................................   1   1   LT-1
.........................cacagcuuucuugaacuuuUuu...........................................................................................................................................................................................................   2   1   LT-3
.........................cacagcuuucuugaacuuuAuu...........................................................................................................................................................................................................   2   1   JCK-3
.........................cacagcuuucuugaacuuuAuu...........................................................................................................................................................................................................   8   1   JCK-2
.........................cacagcuuucuugaacuuuAuu...........................................................................................................................................................................................................   3   1   JCK-1
.........................cacagcuuucuugaacuuAcuuu..........................................................................................................................................................................................................   1   1   LT-1
..........................acagcuuucuugaacuuA..............................................................................................................................................................................................................   2   1   LT-2
..........................acagcuuucuugaacuuu..............................................................................................................................................................................................................   1   0   LT-2
..........................acagcuuucuugaaUuuu..............................................................................................................................................................................................................   6   1   LCK-3
..........................acagcuuucuugaacuuuU.............................................................................................................................................................................................................   3   1   LT-2
..........................acagcuuucuugaacuuuU.............................................................................................................................................................................................................   4   1   LCK-3
..........................acagcuuucuugaacuuuU.............................................................................................................................................................................................................   1   1   LT-3
..........................acagcuuucuugaacuuuA.............................................................................................................................................................................................................   1   1   LT-1
..........................acagcuuucuugaacuuuUu............................................................................................................................................................................................................   3   1   LT-3
.........................................uuucuGucuuucuguuua...............................................................................................................................................................................................   1   1   LT-2
..........................................................................................gcucaagaaagcugugg...............................................................................................................................................   1   0   JCK-2
..........................................................................................gcucaagaaagcuguggga.............................................................................................................................................   1   0   LT-3
..........................................................................................gcucaagaaagcugugggag............................................................................................................................................   2   0   JCK-3
..........................................................................................gcucaCgaaagcugugggag............................................................................................................................................   1   1   JT-3
..........................................................................................gcucaagaaagcugugggag............................................................................................................................................   3   0   JT-2
..........................................................................................gcucaagaaagcugugggag............................................................................................................................................   7   0   JT-3
..........................................................................................gcucaagaaagcugugggag............................................................................................................................................   2   0   JCK-1
..........................................................................................gcucaagaaagcugugggaga...........................................................................................................................................   7   0   LCK-3
..........................................................................................Acucaagaaagcugugggaga...........................................................................................................................................   1   1   JCK-1
..........................................................................................gcucaagaaagcugugggaga...........................................................................................................................................   17  0   JCK-3
..........................................................................................gcucaagaaagcugugggaga...........................................................................................................................................   17  0   JCK-2
..........................................................................................gcucaagaaagcugugggaga...........................................................................................................................................   5   0   LT-3
..........................................................................................gcucaagaaagcugugggaga...........................................................................................................................................   1   0   LT-1
..........................................................................................gcucaagaaagcugugggaga...........................................................................................................................................   13  0   JCK-1
...........................................................................................cucaagaaagcuguggga.............................................................................................................................................   2   0   JCK-3
...........................................................................................cucaagaaagcuguggga.............................................................................................................................................   1   0   JCK-2
...........................................................................................cucaagaaagcugugggag............................................................................................................................................   1   0   JCK-3
...........................................................................................cucaagaaagcugugggag............................................................................................................................................   4   0   JT-3
...........................................................................................cucaagaaagcugugggag............................................................................................................................................   1   0   LT-3
...........................................................................................cucaagaaagcugugggag............................................................................................................................................   6   0   JT-2
...........................................................................................cucaagaaagcugugggag............................................................................................................................................   4   0   JCK-2
...........................................................................................cucaagaaagcugugggag............................................................................................................................................   2   0   JCK-1
...........................................................................................cucaagaaaAcugugggaga...........................................................................................................................................   1   1   LT-2
...........................................................................................cucaagaaagcugugggaga...........................................................................................................................................   23  0   LT-3
...........................................................................................cucaCgaaagcugugggaga...........................................................................................................................................   1   1   JCK-2
...........................................................................................cucaagaaagcugugggaga...........................................................................................................................................   6   0   JT-2
```

Mature — uuccacagcuuucuugaacu

Star — cucaagaaagcugugggaga

uuugcaaguccuggucaugcuuuuccacagcuuucuugaacuuucuuucuuucuguuuagucucuuuuuuaagauugaacaccauugaaagcucaagaaagcugugggagaauauggcaaauuugggauuuuaauuuuuuuauuuuuguuuuuauuuuuuauuuuuccuucaacuacucuucagcugaugaugauguugcacacagaaagaaagaaagaaagaaagaaagagagaucucagaagcuaaau			
cucaagaaagcugugggaga	21	0	LT-2
cNcaagaaagcugugggaga	2	1	JCK-3
Uucaagaaagcugugggaga	1	1	JCK-2
cucaaUaaagcugugggaga	3	1	JCK-3
cucaagaaagAugugggaga	1	1	JCK-2
cucaagaaagcugugggaga	86	0	JCK-1
cucaagaaagcugugggagC	2	1	JCK-2
cucaagaaagcugugggaga	112	0	JCK-3
cucaagaaagcugugggaga	19	0	LT-1
cucaagaaagcugugggaga	8	0	JT-3
cucaagaaagcugugggaga	111	0	JCK-2
cucaagaaagcugugggagC	2	1	JCK-3
cucaagaaagcugugggagaC	2	1	JCK-2
cucaagaaagcugugggagaC	1	1	JCK-1
cucaagaaagcugugggagaU	1	1	JCK-3
cucaagaaagcugugggagaUu	1	1	LT-3
ucaagaaagcugugggaga	3	0	JCK-2
ucaagaaagcugugggaga	1	0	JCK-3
ucaagaaagcugugggaga	1	0	JCK-1
aagaaagcugugggagaauauggc	1	0	LT-3
aagaaagcugugggagaauauggc	2	0	JT-3
aagaaagcugugggagaauauggc	4	0	JCK-1
aagaaagcugugggagaauauggc	10	0	JCK-2
aagaaagcugugggagaauauggc	4	0	JT-2
aagaaagcugugggagaauauggc	1	0	LT-1
aagaaagcugugggagaauauggc	10	0	JCK-3
aaagcugugggagaauauggc	1	0	JCK-3
aaagcugugggagaauauggc	2	0	JCK-2
aagcugugggagaauauggc	3	0	LT-1
aagcugugggagaauauggc	1	0	JCK-3
aagcugugggagaauauggc	1	0	JCK-2
aagcugugggagaauauggc	3	0	LT-2
aagcugugggagaauauggca	8	0	JT-2
aagcugugggagaauauggca	6	0	JCK-2
aagcugugggagaauauggca	5	0	JT-3
aagcugugggagaauauggca	3	0	LT-3
agcugugggagaauauggc	3	0	JCK-2
ugugggagaauauggcaaauu	1	0	JCK-2
ugugggagaauauggcaaauu	1	0	JCK-3
